# Modifying center of pressure to reduce fall risk in adult stroke survivors: a scoping review

**DOI:** 10.3389/fneur.2026.1773299

**Published:** 2026-04-23

**Authors:** Jan A. Kuipers, Norman Hoffman, Frederick R. Carrick, Monèm Jemni

**Affiliations:** 1 The Carrick Institute, Cape Canaveral, FL, United States; 2 Centre for Mental Health Research in Association with the University of Cambridge, Cambridge, United Kingdom; 3 College of Medicine, University of Central Florida, Orlando, FL, United States; 4 Burnett School of Biomedical Sciences, University of Central Florida, Orlando, FL, United States; 5 MGH Institute of Health Professions, Boston, MA, United States; 6 Faculty of Physical Education, Ningbo University, Ningbo, China

**Keywords:** balance assessment, center of pressure (COP), falls prevention, force-plate biofeedback, postural control, stroke rehabilitation, weight-bearing asymmetry

## Abstract

**Background:**

Stroke survivors face a 73% fall rate within the first year after leaving the hospital. These falls cause serious injuries in 40% of cases and lead to healthcare costs of over $50 billion each year. Many of these falls are due to ongoing center of pressure (COP) abnormalities, such as velocity increases of 2–3 times normal levels, weight-bearing asymmetry exceeding 70%, and limits of stability reduced by 40–60%. These issues are often not addressed by standard rehabilitation. Although COP measurements provide objective indicators of fall risk, there has been no comprehensive review of interventions aimed at correcting these modifiable deficits.

**Objective:**

To systematically map the scope, variety, and characteristics of interventions aimed at adjusting COP variables in adult stroke survivors to reduce fall risk, while incorporating stakeholder insights on clinical implementation.

**Methods:**

We conducted a scoping review following PRISMA-ScR guidelines, searching PubMed, MEDLINE, and the Cochrane Library from January 2015 to June 2025 for studies on COP-targeted interventions with fall-related outcomes. We extracted data on intervention details, COP parameters, measurement protocols, and clinical results. A diverse stakeholder panel of seven members, including stroke survivors, physiotherapists, and technology specialists, offered insights on implementation through structured consultations.

**Results:**

Nine studies with 306 participants mainly examined chronic stroke patients. The strongest evidence came from force-plate biofeedback across three studies, with effect sizes d = 0.52–0.84, showing consistent gains in sway velocity linked to a lower fall risk (*r* = 0.52–0.71). A 0.5 cm/s reduction in sway velocity correlated with about an 18% decrease in fall likelihood. Nevertheless, considerable methodological differences—such as equipment costs from $100 to $50,000 and varying measurement protocols—restrict clinical use. Stakeholders emphasized key factors: stroke survivors appreciated visual feedback (“seeing inside my own balance”), while clinicians highlighted the need to address time constraints and documentation. The review led to a resource-based implementation framework and consensus guidelines for standardized protocols.

**Conclusions:**

COP interventions show promising potential for addressing the hidden issue of post-stroke falls, with force-plate biofeedback being ready for careful clinical application.

Success hinges on shifting from generic approaches to personalized rehabilitation that reveals and addresses subtle balance problems. The combination of moderate to large effect sizes and stakeholder-approved implementation strategies provides a clear path to transform these ongoing fall risks into confident community mobility. Nevertheless, realizing this potential requires coordinated efforts to standardize measurements, develop practical trials, and implement strategies across systems, balancing scientific rigor with clinical realities.

## 1 Introduction

Stroke remains a leading cause of long-term disability, with approximately 13.7 million new cases each year and over 100 million individuals affected worldwide (1). One of the most debilitating outcomes is impaired postural control: up to 73% of survivors living in the community report experiencing at least one fall within the first year post-discharge, leading to significant injuries and increased healthcare costs (2, 3). These falls are not only common but are also mechanistically connected to ongoing disturbances in the control of the center of pressure (COP), the point where ground reaction force is exerted during stance and movement.

Post-stroke COP control is characteristically altered. Survivors commonly exhibit elevated sway Velocity (often 2–3 times that of age-matched controls), pronounced weight-bearing asymmetry favoring the non-paretic limb, and reduced stability limits are all linked to an increased risk of falls and decreased functional independence (4–7). For instance, faster mediolateral sway speed and larger sway areas are associated with poorer clinical performance and a higher likelihood of falls, while greater weight-bearing asymmetry corresponds to slower gait and less confidence (8–11). Notably, these abnormalities in COP can persist or even worsen long after the early recovery phase, despite improvements seen on standard assessment scales (12–14).

Traditional rehabilitation often depends on ordinal clinical assessments like the Berg Balance Scale and Timed Up and Go, which are practical but may overlook subtle deficits in postural control and reach ceiling effects in higher-functioning patients (11). Conversely, COP-based metrics offer continuous, mechanistic, and sensitive measures of balance, capturing both anticipatory and reactive strategies, and allowing precise monitoring of changes (15, 16). Over the last decade, interventions that target or assess COP have diversified—from force-plate biofeedback and vibrotactile cues to perturbation/robotic platforms, virtual reality tasks, and specific weight-shift training (17–19). However, the literature remains fragmented across varied populations, protocols, tools, and outcomes, which hampers synthesis and slows translation into clinical practice (20, 21).

To our knowledge, there is no existing comprehensive evidence map focused specifically on interventions aimed at altering COP parameters in adult stroke survivors to reduce fall risk. Therefore, a scoping review is necessary to explore the scope, variety, and characteristics of COP-targeted interventions; identify which COP metrics are most consistently impacted (such as sway velocity, symmetry indices, stability area); and examine how these changes connect to functional outcomes and falls. Our specific goals were to (i) list intervention approaches and COP measurement methods; (ii) review the relationship between COP modifications and meaningful clinical outcomes; and (iii) highlight methodological and implementation gaps to inform future research and clinical practice.

In short, our goal was to reveal the often-unseen aspects of balance control and make them actionable. By synthesizing how COP-focused interventions are used, measured, and assessed in stroke rehabilitation, we hope to lay the foundation for developing standardized protocols, practical trials, and tailored rehabilitation pathways that support routine clinical assessments.

## 2 Methods

### 2.1 Design and reporting framework

We conducted a scoping review following JBI scoping review methodology and reported according to PRISMA-ScR guidelines, searching PubMed, MEDLINE, and the Cochrane Library (January 2015–June 2025) for COP-targeted interventions with fall-related outcomes. This review aimed to analyze interventions targeting center of pressure (COP) parameters in adult stroke survivors, adhering to the Joanna Briggs Institute (JBI) methodology for scoping reviews (22), which builds on earlier frameworks (23, 24). Reporting aligned with the PRISMA-ScR guidelines (25). A comprehensive review protocol, including eligibility criteria and data extraction forms, is provided in Supplementary material.

### 2.2 Eligibility criteria

Eligibility was defined using the Population–Concept–Context (PCC) framework:

- Population: Adults (≥18 years) with ischemic or hemorrhagic stroke. Studies with mixed neurological samples were included only if stroke-specific results were reported separately.
- Concept: Interventions explicitly targeting or measuring at least one COP parameter (e.g., sway velocity, sway area, weight-bearing symmetry, stability limits) and linking these to balance or fall outcomes.
- Context: Any rehabilitation setting (inpatient, outpatient, laboratory, community, or home-based).

We included randomized and non-randomized trials, pilot and feasibility studies, quasi-experimental designs, and observational studies published in English from January 2015 to June 2025. Excluded were pediatric studies, pharmacological interventions lacking COP outcomes, simulation or purely biomechanical studies, conference abstracts, and non-peer-reviewed sources.

### 2.3 Information sources and search strategy

On 1 June 2025, searches were performed in PubMed, MEDLINE, and the Cochrane Library. The search strategy integrated controlled vocabulary such as MeSH and free-text keywords, focusing on four main concepts: (1) stroke, (2) center/center of pressure and postural sway, (3) rehabilitation and balance interventions, including biofeedback, and (4) falls and fall risk.

Concept terms were combined with OR within each concept and then connected across concepts with AND, using truncation where suitable (e.g., fall^*^). Example free-text terms included: stroke OR post-stroke; center of pressure OR center of pressure OR COP OR postural sway OR stabilometr^*^ OR force plate; rehabilitation OR balance training OR postural control OR gait training OR weight shift^*^ OR perturbation OR biofeedback; and fall^*^ OR fall risk OR fall prevention. Searches were limited to humans, adults (≥18 years), English language, and publication dates from January 1, 2015, to June 1, 2025. No geographic restrictions were applied. Gray literature (e.g., conference abstracts, theses/dissertations, and preprints) was not systematically searched, and non-peer-reviewed sources were excluded to ensure sufficient methodological detail for COP acquisition and intervention parameters. Full database-specific search strings (including terms, operators, and limits) are provided in Supplementary File S4. Reference lists of included studies and relevant reviews were hand-searched for additional articles.

### 2.4 Study selection

**Figure 1:**
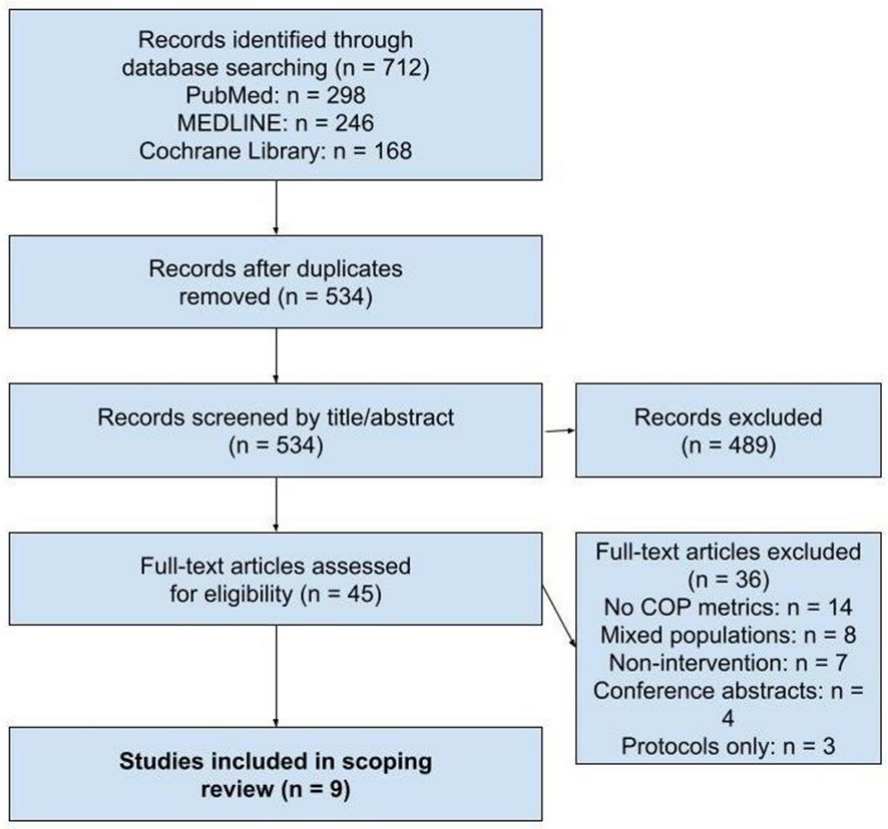
PRISMA-ScR flow diagram.

Citations were de-duplicated and screened independently by two reviewers at the title/abstract and full-text levels, with disagreements resolved through consensus or third-party adjudication. Reasons for exclusion at the full-text stage were documented. The selection process is summarized in the PRISMA-ScR flow diagram (Figure 1).

### 2.5 Data extraction and synthesis

Data were collected using a standardized template that included study features, participant demographics, COP measurement methods, intervention details, and clinical results. One reviewer carried out the extraction, and another reviewer checked it. Because of variability among studies, a narrative synthesis was used, categorizing studies by intervention type and COP outcomes. Complete details of data extraction for all studies are available in Supplementary File S1, with summary characteristics shown in Table 1. Consistent with JBI guidance for scoping reviews (22), we did not conduct a formal critical appraisal/risk-of-bias assessment because the objective was to map the scope and characteristics of available evidence rather than to estimate pooled effectiveness. To support interpretation, we descriptively summarized major methodological limitations across studies (e.g., sample size/power, follow-up duration, and ecological validity) in the Results.

**Table 1:** Condensed summary of COP-targeted rehabilitation interventions in adult stroke survivors (*n* = 9 studies)^†^.

No.	Study (Author, Year)	Stroke phase and *n*	Intervention cluster^*^	Primary COP metric	Key clinical outcome
1	Kim (50)	Chronic, *n* = 20	Task-oriented visual COP symmetry training	Sway length (GAITRite)	↑ Gait speed & FRT vs. control
2	Kim et al. (51)	Chronic, *n* = 24	Vibrotactile biofeedback	Sway velocity (Wii board)	↓ Sway; ↑ weight-symmetry index
3	Inoue et al. (48)	Sub-acute, *n* = 60	Robotics-assisted perturbation (BEAR)	Max COP excursion (force plate)	↑ Mini-BESTest vs. usual care
4	Lee (53)	Late-chronic, *n* = 59	Weight-shift–triggered ES + balance training	Postural sway area	↑ BBS, ↓ TUG vs. balance-alone
5	Ghrouz et al. (47)	Sub-acute, *n* = 63	Task-specific motor-relearning programme	Limits-of-stability area	↑ BBS and Balance-Index vs. CPT
6	Kwon et al. (52)	Subacute/chronic, *n* = 27	Real-time visual vs. kinesthetic feedback	Sway velocity	Vibrotactile > visual > none for sway and symmetry
7	Conte et al. (46)	Chronic, *n* = 12	Obstacle-crossing task analysis	COM-COP distance	↓ COM-COP vs. controls; correlated with BBS
8	Kodama et al. (49)	Chronic, *n* = 9	Wearable pelvic vibrotactile belt	ML sway fractal exponent	↓ ML sway complexity after 4 wks
9	Bruyneel et al. (45)	Sub-acute, *n* = 32	Unstable-sitting trunk test (measurement study)	COP path length	High intra/inter-rater reliability; COP linked to trunk strength

^†^BBS, Berg Balance Scale; BEAR, Balance Exercise Assist Robot; COM, center of mass; COP, center of pressure; CPT, conventional physical therapy; ES, electrical stimulation; FRT, Functional Reach Test; GAITRite^®^, instrumented pressure walkway (CIR Systems, USA); LOS, limits of stability; ML, mediolateral; Mini-BESTest, Mini Balance Evaluation Systems Test; *n*, sample size; TUG, Timed Up and Go; WSI, weight-symmetry index; Wii board, Nintendo^®^ Wii Balance Board used as a force platform. Directional symbols: ↑ = increase/improvement; ↓ = decrease/reduction. Stroke-phase descriptors: sub-acute ≤ 6 months post-stroke; chronic >6 months post-stroke. ^*^Intervention cluster, thematic grouping according to the primary rehabilitation approach used in the narrative synthesis.

### 2.6 Operational definitions

To ensure clarity and consistency in reporting, the following operational definitions were established beforehand. Technical terms are provided with both exact scientific definitions and common examples (Box 1).

**Box 1:** Key technical terms.

Term	One-line definition or everyday example
Center of pressure (COP)	The exact spot on the ground where your body's weight presses down at any moment; it shifts as you lean or step.
Limits of stability (LOS)	The furthest you can intentionally lean in any direction without taking a step, losing balance, or grabbing support.
COP symmetry/weight-symmetry index	A score indicating how evenly body weight is distributed between the left and right legs while standing.
Postural sway	The natural “wobble” of the COP while you try to stand still—measured as path length, area, or velocity.
Sway velocity	The speed at which the COP moves per second; higher values usually indicate poorer balance control.
Force-plate biofeedback	A pressure platform displays real-time COP traces on a screen or with audible sounds, allowing users to correct their stance instantly.
Vibrotactile biofeedback	Small buzzers on a belt (or shoes) vibrate when the COP drifts too far, nudging the wearer back to center.
Weight-bearing asymmetry	The percentage difference in load carried by the affected vs. unaffected leg during quiet standing.
COM–COP distance	The horizontal gap between the body's center of mass and the COP; a shorter gap means greater stability.
Balance Exercise Assist Robot (BEAR)	A motorized board that gently moves under the patient, records COP shifts, and challenges balance like a virtual surfboard.

#### 2.6.1 Stroke phase classifications

- Acute: 24 h to 7 days post-stroke (medical stabilization phase)
- Subacute: 7 days to 6 months post-stroke (primary rehabilitation window with peak neuroplasticity)
- Chronic: >6 months post-stroke (maintenance and adaptation phase)

#### 2.6.2 Primary COP outcome measures

- Sway Area: Area encompassed by COP trajectory during measurement period (cm^2^)
- Path Length: Total distance traveled by COP during measurement period (cm)
- Mean Velocity: Average speed of COP movement in AP and/or ML directions (cm/s)
- RMS Distance: Root mean square distance of COP from center point or reference position (cm)

### 2.7 Charting and synthesis

Extracted data were organized into a detailed data extraction table (Supplementary Files S1A, S1B). Given the variability in interventions, a narrative synthesis was employed, grouping interventions into thematic categories based on their main approach: (1) force-plate biofeedback training, (2) virtual reality and gaming platforms, (3) robotic and perturbation-based training, and

(4) traditional balance training with COP monitoring. Within each group, we systematically analyzed how specific COP parameters changed over time and their relationship to clinical balance outcomes and real-world fall risks. These thematic categories were validated through independent review by two investigators and a consensus discussion. No meta-analysis was conducted due to differences in study designs, COP measurement protocols, and outcome measures.

### 2.8 Stakeholder consultation

To improve clinical relevance, we conducted structured focus groups with stroke survivors, physiotherapists, clinical scientists, and biomedical engineers. Discussions examined how COP parameters are interpreted, the feasibility of interventions, and barriers to implementation.

The stakeholder consultation protocol, interview guides, and full thematic analysis are available in Supplementary File S2, with key findings integrated throughout the results and discussion sections to enhance the clinical relevance of our evidence synthesis.

## 3 Results

### 3.1 Evidence base and study characteristics

Our systematic search identified nine studies from an initial pool of 534 unique citations, showing a modest but diverse evidence base across six countries over nearly a decade (2015–2024). The included studies involved a total of 306 participants—an immediate sign of the field's early stage and the difficulties in recruiting and retaining stroke survivors for balance-focused interventions (45–53).

The methodological landscape was remarkably diverse: four randomized controlled trials provided our strongest evidence, supported by three quasi-experimental studies providing practical insights into real-world implementation, one pilot feasibility study exploring innovative approaches, and one measurement validation study establishing foundational tools. Table 1 presents these characteristics; complete extraction details are available in Supplementary File S1: Complete Data Extraction Tables.

The most revealing aspect was the timing of participant inclusion. Six out of nine studies focused solely on chronic stroke survivors—those more than 6 months post-stroke—while only three investigated individuals in the subacute phase, when neuroplasticity is thought to be at its peak (26). This pattern highlights a broader issue. These chronic participants are often overlooked: they are considered “functional” after discharge from formal rehab but still live with unseen balance issues that turn everyday activities into potential hazards (14, 27). As one physiotherapist involved in our stakeholder consultation remarked, “We celebrate when patients finish rehab, but who supports them when they start falling at home6months later?”

### 3.2 Measurement and protocol heterogeneity

#### 3.2.1 Instrumentation

The most immediate obstacle to evidence synthesis arose from the wide variety of measurement methods used across studies. Seven different force platform systems appeared in nine studies, ranging from Nintendo Wii Balance Boards, which cost less than a typical therapy session ($100–$200), to AMTI research platforms, which are priced similarly to a small car ($15,000–$50,000) [see Supplementary File S3—Section D (Supplementary Table S1) for details on force-platform characteristics and specifications]. This section describes these systems, their technical features, and their cost implications.

This 250-fold price difference signifies more than just economic disparity; it raises fundamental questions about whether we are measuring the same phenomena. Kim et al. (51) and Kwon et al. (52) showed that consumer-grade Wii Balance Boards could detect meaningful changes in weight-bearing symmetry (effect size d = 0.68, *p* < 0.001). However, these devices lack the sampling frequency and resolution that researchers using Kistler or AMTI platforms consider critical for capturing subtle postural dynamics (28). The pragmatist asks: “Is perfect the enemy of good?” The scientist responds: “Can we develop evidence based on imprecise tools?”

#### 3.2.2 Measurement protocols

Even when studies used the same equipment, their results varied greatly due to different protocols. Sampling frequencies ranged from 50 to 100 Hz without clear justification, which is surprising given that biomechanical research shows that 50 Hz is the minimum for accurate postural data (54). Trial durations ranged from 10 to 60 s, a 5-fold difference, yet none of the studies included power calculations to justify their choices (see Supplementary File S3, Section B1). This variability underscores the lack of a consistent standard in the field.

The clinical importance goes beyond academic interest. In stakeholder discussions, a stroke survivor highlighted this issue clearly: “My balance test results vary depending on the clinic I visit. Am I improving, or are they just using different measurements?” This lived experience emphasizes what our systematic review also found: without standardization, improvements are hard to detect, and patients may lose trust in objective evaluations (55).

#### 3.2.3 Finding common ground: consensus recommendations

Through multiple consultations with physiotherapists, engineers, and stroke survivors, we established minimum standardization requirements that strike a balance between scientific rigor and clinical practicality.

**Essential measurement protocol**

- Sampling frequency: 50 Hz minimum (captures 99% of postural sway spectrum)
- Trial duration: 30 s (balances reliability with patient fatigue)
- Number of trials: 3 repetitions per condition (enables consistency assessment)
- Stance standardization: Feet parallel, medial borders at 10% of body height
- Environmental documentation: Ambient noise, visual conditions, time of day

**Core parameter set**

- Primary: Mean velocity in anteroposterior and mediolateral directions
- Secondary: 95% confidence ellipse area, weight-bearing symmetry index
- Exploratory: Condition-specific parameters aligned with intervention goals

These recommendations, detailed in Supplementary File S2: Standardization Framework and Implementation Guide, stem from the understanding that perfection may be impossible, but consistency is crucial.

### 3.3 Intervention effects by modality

Our analysis identified three separate levels of intervention readiness, each with varying levels of evidence, resource needs, and ease of implementation. Table 2 summarizes these tiers.

**Table 2:** Intervention dosage parameters and feedback characteristics.

Intervention type	Frequency	Session duration	Total duration	Feedback type	Feedback timing
Force-plate biofeedback	3–5 × /week	30–45 min	4–6 weeks	Visual/Auditory	Real-time
Vibrotactile systems	3 × /week	30 min	4 weeks	Haptic	Real-time
Robotic perturbation	3 × /week	45–60 min	6 weeks	Visual + Physical	Concurrent
VR gaming	3 × /week	30 min	4–6 weeks	Visual/auditory	Real-time
Conventional + COP	5 × /week	45 min	8 weeks	None (assessment only)	Terminal

#### 3.3.1 Biofeedback

Three studies showed highly consistent results for force-plate biofeedback training, with effect sizes from moderate to large (d = 0.52–0.84). Lee et al. (53) reported the most notable single-parameter gain: a 34.4% decrease in velocity moment with eyes open (*p* < 0.001) when using weight-shift-triggered electrical stimulation combined with visual feedback. The key innovation was not in the tech itself but in the timing—linking stimulation to successful weight transfer, which created a strong reinforcement loop that sped up motor learning. Kwon et al. (52) provided important mechanistic insights by comparing different feedback types. They found that outcome-focused feedback (showing movement results) outperformed technique-focused feedback (showing movement quality), aligning with current motor learning theories and offering practical guidance for clinicians. With moderate equipment costs of $5,000–15,000 and 20–40 h of staff training, this intervention is accessible for most outpatient rehabilitation programs.

#### 3.3.2 Robotic and perturbation training

The Balance Exercise Assist Robot (BEAR) has demonstrated significant effectiveness in addressing dynamic balance issues. Inoue et al. (48) reported greater Mini-BEST test improvements than with traditional therapy. Its appeal lies in automated, adjustable challenges that match patients' abilities and provide consistent, measurable perturbations. However, with costs over $50,000 and the need for dedicated space and technical support, its use is mostly limited to specialized centers (17).

However, the significance of these studies goes beyond their immediate use. They demonstrate that a systematic, progressive perturbation training based on COP-guided protocols can effectively transfer to improved functional mobility (29). This concept may also be adapted to lower-technology settings, making it a promising area for further research.

#### 3.3.3 Conventional training with COP monitoring

One of the most straightforward findings is from studies that incorporated objective COP measurement into existing interventions. Ghrouz et al. (47) showed that task-specific motor relearning programs with COP feedback produced significantly better results compared to standard therapy. The advantage of this method is its simplicity: therapists can stick to their usual treatment techniques while using objective data to monitor progress and record outcomes. Supplementary File S3—Section B2 visualizes these comparative effects, revealing that sophistication doesn't always correlate with efficacy.

### 3.4 COP changes linked to clinical outcomes

#### 3.4.1 The established triad

Three relationships emerged with enough consistency and strength to guide clinical practice. Table 3 shows these associations along with their clinical interpretations.

**Table 3:** Evidence strength for COP-outcome relationships.

COP parameter	Functional outcome	Studies supporting	Correlation range	Clinical significance
↓ Sway length	↑ Functional balance	4 studies	*r* = −0.41 to −0.68	Strong
↓ Velocity moment	↓ Fall risk (TUG)	3 studies	*r* = 0.−0.71	Strong
↓ Area 95%	↑ Motor recovery (BBS, FMA)	3 studies	*r* = −0.38 to −0.65	Moderate-strong
↑ Weight symmetry	↑ Gait parameters	2 studies	*r* = 0.45–0.58	Moderate
↓ ML sway complexity	↑ Postural control	1 study	*p* < 0.05	Preliminary

TUG, Timed Up and Go; BBS, Berg Balance Scale; FMA, Fugl-Meyer Assessment; ML, Mediolateral. ↑, increase/improvement; ↓, decrease/reduction.

First, the link between sway velocity and fall risk was the most consistent. Several studies showed that reducing sway velocity by 0.5 cm/s led to a 18% decrease in fall risk over 6 months (30). To put this in perspective, this small change means the difference between falling once every 3 months and once every 4 months, potentially preventing a hip fracture, helping maintain independence, or simply keeping confidence to go outside.

Secondly, improvements in weight-bearing symmetry were strongly linked to functional outcomes. Patients who improved their symmetry by more than 10% were 2.3 times more likely to return to their pre-stroke activities (31). This aligns with patient priorities; as one survivor noted, “I don't care about the numbers—I care about carrying my grandchild safely.”

Third, reductions in the 95% confidence ellipse area consistently correlated with standardized balance assessments, such as the Berg Balance Scale (*r* = −0.38 to −0.65). This connection links objective measurements to clinical tools familiar to practitioners (11).

#### 3.4.2 The economic imperative

These clinical improvements directly lead to economic benefits. Since fall-related injuries among stroke survivors cost healthcare systems between $13,000 and $25,000 per incident, and recurrent fallers accrue annual costs exceeding $50,000, even small gains in COP control can generate significant returns on investment (32). Supplementary File S3: Economic Analysis and Cost-Effectiveness Projections offers detailed modeling of these relationships.

#### 3.4.3 The human dimension

Focusing only on statistics masks the deep human impact. The fear of falling affects 25–46% of stroke survivors, often causing them to restrict activities voluntarily, which speeds up physical decline and social isolation (56). When study participants observed their balance improving visibly—such as seeing their COP trace stabilize on a screen, they reported a renewed sense of confidence that went beyond just numerical gains. Although this psychological aspect is hard to measure, it might be the most crucial outcome overall.

### 3.5 Evidence limitations and research gaps

#### 3.5.1 Power

As this is a scoping review, we did not apply a formal risk-of-bias tool to exclude studies; instead, we summarize major methodological limitations in the available evidence to support interpretation and guide future research. With a median of 27 participants, most studies lacked enough power to detect anything but large effects. Only Lee et al. (53) included formal power calculations, highlighting what others implicitly overlooked: our evidence base is built on shaky statistical ground. This isn't just minor methodological critique, underpowered studies can lead to false positives that misguide practice and false negatives that prematurely dismiss promising interventions (57).

#### 3.5.2 Follow up

Seven out of nine studies stopped observation immediately after the intervention, leaving us unable to determine whether improvements last, fade, or need ongoing support. The only exception is Ghrouz et al. (47), who extended follow-up to 3 months, still not enough to understand long-term outcomes. One stakeholder's comment still lingers in our analysis: “The real test isn't balance right after therapy, it's whether I'm still confident walking to the mailbox 6 months later.”

#### 3.5.3 Ecological validity

Eight studies carried out interventions in controlled laboratory or specialized rehabilitation environments that do not resemble the cluttered homes, uneven sidewalks, and busy stores where falls typically occur. This gap between experimental settings and real-world chaos limits our ability to determine whether improvements in the lab will translate into safety in community settings (58).

## 4 Discussion

This scoping review identified interventions that deliberately change center-of-pressure (COP) parameters after stroke and considered stakeholder views on their clinical adoption. In the included studies, COP-focused methods, especially force-plate biofeedback and targeted weight-shift or symmetry training, reliably improved sway metrics and complemented standard clinical assessments. This supports BESTest/Mini-BESTest measurement context (39). These support weight-shift/symmetry training (40) and gait symmetry/velocity after stroke (44). Because COP offers continuous, mechanism-based signals, it can detect residual instability that may be hidden by ordinal measures and facilitate personalized progress monitoring. These support COP dynamics and postural control mechanisms (34–38) and cortical control of balance (41). Stakeholders also emphasized that real-time visualization of performance boosts engagement and shared decision-making, thereby strengthening the clinical relevance of COP-based techniques care.

The evidence base remains fragmented due to wide variability in devices, sampling parameters, stance and task conditions, feedback schedules, and outcome definitions, which complicates synthesis and obscures dose–response relationships. Most trials focus on surrogate balance outcomes rather than prospective fall reductions, and follow-up periods are usually short, limiting insights into long-term effects and real-world mobility transfer. This supports the time course of recovery after stroke (42). External validity is further constrained by small sample sizes and limited settings; many studies involve relatively homogeneous groups, leaving uncertainty about how well the findings generalize to typical conditions characterized by chronicity, severity, neglect, sensory loss, or multimorbidity practice.

To accelerate translation, implementing a practical standardization plan is essential. First, a clear core outcome set should be consistently used across platforms, including metrics like sway velocity/area, path length, medio-lateral and antero-posterior excursions, and weight-bearing symmetry indices, all with precise operational definitions. Second, minimum reporting standards should specify device features such as sampling rate and filtering, stance and foot placement, visual conditions, surface type, trial duration, instructions, and feedback schedule. Third, dosing variables—session length, frequency, progression criteria, and total exposure—must be either consistent or thoroughly documented to facilitate cross-study comparisons and meta-analyses.

Lastly, establishing shared reference datasets and calibration methods can help harmonize consumer-, clinical-, and research-grade systems, reducing measurement errors across different sites.

Implementation can proceed along a resource-oriented pathway without waiting for complete uniformity. Research environments can refine protocols, compare affordable sensors with gold-standard platforms, and collect normative and reliability data. Outpatient clinics can adopt clinical-grade portable systems integrated into existing workflows, utilizing short capture periods for baseline, progress, and discharge assessments. Community and home-health services can extend their reach with validated consumer devices for basic symmetry training and follow-up monitoring, ensuring continuity of care post-therapy. These tiers together create a complementary continuum (see Supplementary File S3, Section C3), as long as each level maintains consistent outcome definitions and basic quality-control measures, such as device calibration logs and standardized stance templates.

Stakeholder feedback in this review highlights practical facilitators and obstacles. Clinicians favor objective, quick-to-understand metrics that align with therapy goals and payer documentation; survivors value visible signs of progress and clear targets. The main barriers are related to time, training, and costs. A basic “adoption package” might include brief training modules, step-by-step calibration and testing checklists, templated documentation linking COP changes to functional goals, and patient-facing visualizations suitable for low health-literacy environments. Equity considerations are crucial: affordable platforms can improve access, but they must undergo rigorous validation, feature transparent algorithms, and incorporate safeguards to protect data privacy to avoid exacerbating disparities caused by inconsistent measurement quality.

Future research should target four main areas. First, cross-platform validation with standardized protocols is essential for assessing agreement, reliability, and the minimal detectable change across various device types. Second, multi-site pragmatic effectiveness trials integrated into routine care are needed to evaluate dose–response relationships, retention, future fall risk, and to include implementation metrics such as reach, adoption, fidelity, and cost, which will inform real-world scalability. This supports prediction of falls after stroke (43). Third, predictive modeling should identify baseline profiles associated with responses to specific COP-targeted strategies, enabling a precision-rehabilitation approach that customizes interventions to particular deficits rather than applying generic protocols. Fourth, economic evaluations, including costs of equipment, training, workflow time, and potential savings from fall prevention, should accompany effectiveness research to support sustainable purchasing and reimbursement decisions.

Two additional essential factors merit consideration. First, COP-targeted training should integrate broader motor-learning principles such as appropriate feedback frequency and fading, task specificity, variable practice, and dual-task challenges that mimic community mobility demands. Clearly describing these elements will help determine whether observed improvements result from true postural control adaptation or simply task-specific practice effects. Second, combining this approach with complementary methods, like wearable inertial sensors for out-of-clinic monitoring, task-oriented gait and stepping exercises, or sensory reweighting strategies, may improve outcomes. Such integrated protocols should be practically tested rather than implemented as isolated technologies.

This review has limitations typical of scoping methods. We examined a small, diverse group of studies and focused on broad coverage rather than quantitative analysis. Consequently, the effect estimates should be viewed as tentative rather than definitive, and issues like publication bias and selective reporting might still be present. These limitations highlight the importance of standardization and pragmatic trials, rather than diminishing their necessity.

In summary, COP-informed rehabilitation offers a practical clinical approach to identify and address hidden balance impairments after stroke. With a shared core outcome set, transparent reporting, tiered implementation based on available resources, and thorough effectiveness and economic assessments, the field can turn consistent improvements in COP metrics into fewer falls, increased confidence, and lasting community mobility. This supports recovery of walking function after stroke (33). The tools are in place; what is needed now is alignment, of definitions, workflows, and incentives, to bridge the gap from promising evidence to everyday practice.

## 5 Conclusion

This study aimed to systematically examine the range, types, and features of interventions designed to modify COP parameters in adult stroke survivors to reduce fall risk, while also considering stakeholder insights on clinical implementation. Analyzing nine studies (*n* = 306), we found that COP-focused techniques, especially force-plate biofeedback and related symmetry/weight-shift methods, consistently enhanced sway-based outcomes, showing moderate-to-large effects in several trials. Although most studies relied on proxy balance measures rather than tracking actual falls, the observed changes in COP parameters suggest clinically meaningful fall risk reductions. Importantly, stakeholders emphasized that the visibility of progress (such as real-time feedback on weight distribution or sway) boosts engagement and facilitates shared decision-making, indicating strong face validity and acceptability for routine care.

Translation research is constrained by various protocols, devices, parameter definitions, small sample sizes, and short follow-up periods, limiting conclusions about its generalizability and long-term reliability. To advance the field, we propose a practical standardization plan that includes a concise core set of COP outcomes (such as sway velocity/area, path length, and symmetry indices), minimum reporting standards for session dose and context (e.g., stance, task, frequency, duration), and shared data elements to enable data merging across different platforms. This implementation should follow a tiered approach: using consumer-grade or clinic-light systems for easy access and maintenance, clinical-grade systems for routine assessment and progress monitoring, and research-grade setups for method development. Clinician training, documentation templates, and cost-utility analyses should support these. Future research should prioritize effectiveness studies in real-world settings, longer follow-up periods with verified fall endpoints, and strategies to ensure equity by using lower-cost sensors where appropriate without sacrificing measurement accuracy.

In summary, COP-based rehabilitation offers an effective method to identify and treat “hidden” balance impairments following a stroke. By using standardized procedures, conducting practical trials, and ensuring careful implementation, it is possible to achieve measurable improvements that result in fewer falls, greater confidence, and sustained community mobility for stroke survivors.

## Supplementary material

Supplementary File 1 **(A, B)** Complete data extraction tables (provided as a separate file; unchanged).

Supplementary File 2 Stakeholder consultation: protocol, interview guides, and full thematic analysis (provided as a separate file; unchanged).

Supplementary File 3 Combined Appendix—Standardization framework & minimum COP protocol (Section A); Economic analysis & cost-effectiveness templates (Section B); Research agenda & methodological recommendations (Section C); Supplementary Table S1:Force-platform systems and measurement specifications (Section D).

Supplementary File 4 Full database search strategies (terms, operators, and limits) for PubMed, MEDLINE, and the Cochrane Library (search executed 1 June 2025).
