# Supplementary material for: Modifying center of pressure to reduce fall risk in adult stroke survivors: a scoping review
Source: Front Neurol. 2026 Apr 23;17:1773299. doi: 10.3389/fneur.2026.1773299 (PMC13149131; doi:10.3389/fneur.2026.1773299)
Supplement: Supplementary File 1 — (A, B) Complete data extraction tables (provided as a separate file; unchanged). [file Table_1.docx]

**Supplement S1 A. Extraction data, summary of COP-Targeted Rehabilitation Interventions in Adult Stroke Survivors**

| **Paper** | **Study Design and Setting** | **Population characteristics** | **Intervention Details** | **Targeted COP Measurements** | **Comparator or control condition** | **Outcome measures** | **Key Results** |
| --- | --- | --- | --- | --- | --- | --- | --- |
| The effects of symmetric center of pressure displacement training with feedback on the gait of stroke patients  **Jin-Seop Kim**  Journal of Physical Therapy Science  2015 | - Trial type: Controlled trial  - Sample size: 20  - Acute/subacute vs. chronic phase: Chronic phase (at least six months post-stroke)  - Inpatient/outpatient/home: Not explicitly mentioned, but likely outpatient or clinical setting | - Patients with hemiplegia due to stroke  - Chronic stroke patients (at least six months)  - No hemi-spatial neglect or cardiovascular dysfunction  - Could walk independently  - Mini-Mental Status Examination (MMSE-K) score not less than 25 points  - Sex: 6 females, 4 males in the experimental group; 6 females, 4 males in the control group  - Type: 4 hemorrhage, 6 ischemia in the experimental group; 4 hemorrhage, 6 ischemia in the control group  - Age: Experimental group 60.8±6.1 years; Control group 61.4±9.5 years  - Height: Experimental group 160.8±9.7 cm; Control group 164.9±8.4 cm  - Weight: Experimental group 63.8±9.5 kg; Control group 62.2±10.8 kg  - Duration since stroke: Experimental group 9.2±1.8 months; Control group 8.7±2.6 months | - Modality: F-Scan system with visual feedback  - Dosage:  - Session length: 15 minutes  - Frequency: Three times per week  - Total duration: Six weeks | F-Scan system for real-time COP displacement measurement | In feedback training for symmetric pressure, subjects stood on both feet wearing an F-Scan system and performed lateral symmetry training while watching the COP displayed on the monitor in real time. | - Laboratory-based COP metrics: paretic side step length, paretic side stride length, gait velocity (measured using a GAITRite walkway)  - Clinical balance scales: Functional Reach Test (FRT) | - The COP displacement training group significantly improved step length, stride length, gait velocity, and the functional reach test.  - The control group only showed significant improvement in the functional reach test.  - Patients with hemiplegia due to stroke have increased ranges of lateral and anterior/posterior displacement during standing, which increases fall risk.  - The training enabled adjustments in COP displacement velocities to ensure they were not too high, potentially reducing fall risk.  - COP displacement training was more effective than feedback training for even weight distribution in enhancing gait and balance. |
| Effects of Vibrotactile Biofeedback Providing Real-Time Pressure Information on Static Balance Ability and Weight Distribution Symmetry Index in Patients with Chronic Stroke  **Hongjun Kim +1**  Brain Science  2022 · | - Trial type: Randomized crossover study  - Sample size: 24 patients  - Acute/subacute vs. chronic phase: Chronic phase  - Inpatient/outpatient/home: Inpatient (conducted at Y Hospital) | - Patients with chronic hemiplegia  - Chronic stroke for more than 6 months  - Able to stand independently for more than 5 minutes  - Scores less than 40 on the Berg Balance Scale  - Scores greater than 24 on the Mini-Mental State Examination  - Sense of thickness less than 3.61 mm on the Semmes-Weinstein monofilaments test  - Excluded: orthopedic problems, visual impairments, communication issues  - Sex: 18 male, 6 female  - Age: 63.00 (12.31) years  - Height: 163.08 (8.84) cm  - Weight: 59.25 (8.83) kg  - Duration of onset: 15.54 (9.00) months | - Modality: Pressure sensor-based vibrotactile biofeedback, visual biofeedback using a full-length mirror, standing without biofeedback  - Dosage: Each biofeedback condition applied for 1 day with a 24-hour washout period | - Measurement tool: Wii balance board  - Sampling rate: 100 Hz  - Filter: 10 Hz low-pass filter  - Analysis software: Balancia software (version 2.0) | Standing without biofeedback | - Laboratory-based COP metrics: Wii balance board for static balance ability and weight distribution symmetry index  - Clinical balance scales: Berg Balance Scale (BBS) | - Pressure sensor-based vibrotactile biofeedback significantly improves static balance ability and weight distribution symmetry index compared to visual biofeedback and no biofeedback.  - Sway length and sway velocity were significantly lower with vibrotactile biofeedback, indicating better balance control.  - Weight distribution symmetry index was significantly improved with vibrotactile biofeedback, suggesting better weight distribution and reduced risk of falls. |
| Identify the Alteration of Balance Control and Risk of Falling in Stroke Survivors During Obstacle Crossing Based on Kinematic Analysis  **Carmela Conte +6**  Frontiers in Neurology  2019 | - Trial type: Observational study  - Sample size: 12 stroke survivors, 12 healthy controls  - Acute/subacute vs. chronic phase: Chronic phase (implied by moderate to good motor functions)  - Inpatient/outpatient/home: Outpatient setting (implied by hospital ethics committee approval) | - adult men and women aged 57.4 ± 10.8 years (stroke survivors) and 59.3 ± 7.1 years (healthy controls)  - gender-matched  - height-matched (165.3 ± 6.6 cm for stroke survivors, 163.3 ± 5.8 cm for healthy controls)  - mass-matched (64.8 ± 8.5 kg for stroke survivors, 60.9 ± 7.8 kg for healthy controls)  - stroke survivors with a history of stroke (latency since stroke: 17.5 ± 17.1 months)  - lower motor function and balance in stroke survivors compared to healthy controls  - weaker lower limb muscle strength in stroke survivors compared to healthy controls | - Modality: Three-dimensional motion analysis system, force plates, handheld muscle-testing dynamometer  - Dosage: Three successful trials for each obstacle height (10%, 20%, 30% leg length), self-selected walking speed | - Targeted COP parameters: Distance between COM and COP (calculated as RMS during TLP and LLP)  - Measurement tools: Two force plates (AMTI, Watertown, MA, USA) with a sampling frequency of 1 kHz | Twelve age-matched healthy controls | - Laboratory-based COP metrics: COM velocity, COM-COP distance  - Clinical balance scales: Berg Balance Scale (BBS), Fugl-Meyer Assessment (FMA) | - Decreased COM-COP distance in stroke survivors compared to healthy controls during obstacle crossing.  - Positive correlation between COM-COP distance and lower limb muscle strength.  - Positive correlation between COM-COP distance and clinical scales (BBS and FMA).  - Maintaining COM closer to COP increases stability and reduces fall risk. |
| Effects of Balance Exercise Assist Robot training for patients with hemiparetic stroke: a randomized controlled trial  **Seigo Inoue +5** | - Trial type: Assessor-blinded randomized controlled trial  - Sample size: 60 patients  - Phase: Early subacute phase (1 week to 3 months after stroke onset)  - Setting: Inpatient rehabilitation wards at Tokyo Bay Rehabilitation Hospital | - Patients with first-ever hemiparetic stroke  - Aged 40-80 years  - Mean age approximately 64.9 years  - More male patients in the IBT group  - Participants in a rehabilitation setting  - No apparent paralysis in unaffected limbs  - No severe contractures or deformities in lower limbs  - Likely Japanese or residing in Japan | - Modality: Balance Exercise Assist Robot (BEAR) for robotic balance training; Intensive Balance Training (IBT) for supervised balance training  - Dosage: 18 min training session, six times a week, for 2 weeks | - Targeted COP parameters: Maximal movements of the center of pressure from front to back and left to right.  - Measurement tool: Kinetogravicorder G-7100 force plate.  - Measurement conditions: Open-eyed standing position with feet shoulder-width apart. | Conventional inpatient rehabilitation-only (CR group) | - Clinical balance scales: Mini-Balance Evaluation Systems Test (Mini-BESTest), Timed Up and Go test  - Laboratory-based COP metrics: maximal movements of the center of pressure  - Fall incidence or confidence questionnaires: Fall Efficacy Scale-International | - The BEAR and IBT groups showed significant improvements in dynamic balance compared to the CR group.  - The BEAR group demonstrated improvements in reactive postural control.  - The tables provide data on changes in COP movements, which are linked to balance improvements.  - The study suggests that additional balance training with BEAR or IBT improves balance in patients with hemiparetic stroke. |
| Balance Training With a Vibrotactile Biofeedback System Affects the Dynamical Structure of the Center of Pressure Trajectories in Chronic Stroke Patients  **Sonia Julia-Sanchez +5**  Frontiers in Human Neuroscience  2019 · | - Trial type: Non-controlled trial  - Sample size: 9 participants  - Acute/subacute vs. chronic phase: Chronic phase  - Inpatient/outpatient/home: Outpatient setting at the Department of Physical Medicine and Rehabilitation, Tokyo General Hospital | - Chronic stroke patients  - Age range: 50-80 years  - Recruited from the Department of Physical Medicine and Rehabilitation, Tokyo General Hospital  - Gender: Mixed (male and female)  - Type of stroke: Ischemic or hemorrhagic  - Time since stroke: More than 6 months  - Hemiplegic side: Varied (left or right)  - Sensory status: Varied (mild, moderate, severe) | - Modality: Vibrotactile biofeedback system using a Nintendo Wii balance board and vibration motors attached to a belt at the pelvic girdle level.  - Dosage: 45 minutes per session, twice a week, for 4 weeks.  - Session details: Each session included 10 repetitions of balance tasks (1 minute each) with short intervals. | - Measurement tool: Wii Balance board  - Sampling rate: 50 Hz  - Parameters measured: Medio-lateral (ML) and antero-posterior (AP) directions  - Data processing: Savitzky-Golay filter (order 3, length 7)  - Analysis method: Detrended fluctuation analysis (DFA)  - Time scales considered: 0.12 s to 10.86 s | Not mentioned (the paper explicitly states that there was no control group) | Laboratory-based COP metrics: DFA scaling exponents for fast and slow-scale fluctuations in ML and AP directions | - The slow-scale dynamics of ML CoP in stroke patients decreased from pre-training to post-BF training (α = 0.40 ± 0.13 vs. 0.31 ± 0.09).  - The BF training affects postural control strategy used by chronic stroke patients in the ML direction.  - The BF training induced a change in the error correction strategy in ML CoP, suggesting tighter control over body sway.  - The BF training may have a potential to lead the change in the CoP dynamics beyond that of typical rehabilitation. |
| Validity and reliability of center of pressure measures to quantify trunk control ability in individuals after stroke in subacute phase during unstable sitting test  **Anne-Violette Bruyneel +6**  Heliyon  2022 · | - Trial type: Intra-and interrater reliability and validity study  - Sample size: 32 participants  - Phase: Subacute phase (1 week to 3 months post-stroke)  - Setting: Neurorehabilitation department of the Geneva University Hospitals (inpatient/clinical setting) | - adult men and women aged 50 to 75  - mean age: 64.34 years  - gender: 9 women, 23 men  - history of stroke (subacute phase)  - stroke types: hemorrhagic and ischemic  - post-stroke duration: 21 to 86 days  - hemiparesis: 10 right, 22 left  - cognitive function: MMSE score of 25.62  - trunk strength: paretic side 32.29%, nonparetic side 32.11%  - functional reach test: anterior 16.63%, paretic side 10.22%, nonparetic side 10.14%  - mobility: Timed Up and Go test 17.86 seconds | - Modality: Unstable sitting balance test on a seesaw using a force platform (FP)  - Dosage: Two trials per disturbance setting, two sessions with 2-4 hours rest in between, each trial lasting 10 seconds | - Force Platform Model: kin etools 2015, Kicarre company  - Sampling Rate: 100 Hz  - CoP Parameters: length (total, anteroposterior, mediolateral), ellipse area, deltas, mean velocity, maximum velocity, variability  - Filter: 5th Order Butterworth Low Pass Filter with a cut-off at 45 Hz | Not mentioned (the study did not include a comparator or control condition) | - Laboratory-based COP metrics: CoP length parameters, CoP ellipse area, deltas, variability  - Clinical balance scales: Modified Functional Reach Test (MFRT), Balance Assessment in Sitting and Standing (BASSP), Timed Up and Go test (TUG) | - CoP values during anteroposterior perturbation are related to trunk strength.  - CoP length parameters have excellent intra-rater and inter-rater reliability, especially in the mediolateral direction.  - CoP parameters are associated with trunk muscle strength and provide complementary information to other balance tests like MFRT.  - CoP measures are valid for assessing trunk control in individuals after stroke in the subacute phase.  - Reliability is higher for CoP length and velocity compared to other parameters. |
|  |  |  |  |  |  |  |  |
| Effects of Real-Time Feedback Methods on Static Balance Training in Stroke Patients: A Randomized Controlled Trial  **Il-Ho Kwon +3**  Healthcare  2024 · | - Trial type: Single-blind, randomized controlled trial  - Sample size: Initially 30 participants (15 in each group), reduced to 27 due to exclusions  - Acute/subacute vs. chronic phase: Subacute or chronic phase (participants had to maintain standing position for more than 2 min)  - Inpatient/outpatient/home: Inpatient (conducted at Y Hospital in Daejeon Metropolitan City) | - Stroke patients  - Able to independently maintain a standing position for more than 2 minutes  - Berg Balance score of 21-40 (intermediate risk for falls)  - Cognitive ability score of 24 or more on the MMSE-K  - No orthopedic problems, neurological problems other than stroke, or visual impairment  - Hospitalized at Y Hospital in Daejeon Metropolitan City  - Demographic details:  - Sex: KR Group (9 male, 4 female), KP Group (9 male, 5 female)  - Paretic side: KR Group (7 left, 6 right), KP Group (8 left, 6 right)  - Type: KR Group (9 infarction, 4 hemorrhages), KP Group (5 infarction, 9 hemorrhages)  - Disease duration: KR Group (24.92 months), KP Group (19.35 months)  - Age: KR Group (64.53 years), KP Group (63.14 years)  - Height: KR Group (168.62 cm), KP Group (164.29 cm)  - Body mass: KR Group (68.69 kg), KP Group (65.86 kg) | - Modality: Real-time feedback training using a force plate (Model BP400600, AMTI) and a black box with LEDs for feedback display.  - Dosage: 30 minutes per session, five sessions per week, for a total duration of four weeks.  - Additional components: Traditional physical therapy, simple stretches before and after training, external disturbances (front-back, left-right, diagonal). | - Targeted COP parameters: sway length, sway velocity, area 95%  - Measurement tool: force plate (Model BP400600, AMTI)  - Sampling method: force sensing resistors (FSRs) at big toe, head of the first metatarsal bone, head of the fifth metatarsal bone, and heel | Not mentioned (the study did not include a control group) | - Laboratory-based COP metrics: sway length, sway velocity, area 95%  - Clinical balance scales: Berg Balance Scale, Fugl Meyer Assessment for Lower Extremity, Postural Assessment Scale for Stroke, Trunk Impairment Scale  - Fall confidence questionnaire: Fall Efficacy Scale | - Significant improvements in static balance measures (sway length and area 95%) over time.  - Group × time interaction significant for sway length and area 95% with eyes open.  - Both groups showed significant improvements in secondary outcomes related to balance and fall efficacy.  - Real-time visual feedback enhanced balance by providing immediate COP feedback.  - Significant changes in fall efficacy over time in both groups, indicating reduced fall risk. |
| Balance Training with Weight Shift-Triggered Electrical Stimulation for Stroke Patients: A Randomized Controlled Trial  **Kyeongjin Lee**  Brain Science  2023 · | - Trial type: Randomized controlled trial  - Sample size: Initially 60 participants (29 in BT-ESG, 30 in BTG), with 59 completing the study  - Phase: Late chronic phase (more than a year after stroke onset)  - Setting: Inpatient (hospitalized at S Hospital in Seoul, South Korea) | - Chronic stroke patients  - Recruited from S Hospital in Seoul, South Korea  - Understood verbal instructions  - Mini-Mental State Exam score of ≥24  - Could stand independently without assistance  - Brunnstrom motor recovery level ≥4  - Late chronic phase patients with stroke onset more than a year ago  - Age: 66.24 ± 7.01 (BT-ESG), 68.87 ± 7.30 (BTG)  - Height: 163.48 ± 7.46 (BT-ESG), 161.93 ± 10.20 (BTG)  - Weight: 60.50 ± 8.01 (BT-ESG), 60.93 ± 8.64 (BTG)  - Body mass index: 22.60 ± 2.34 (BT-ESG), 23.16 ± 1.81 (BTG)  - Duration of stroke: 14.17 ± 5.87 (BT-ESG), 16.33 ± 5.84 (BTG)  - MMSE: 25.83 ± 1.23 (BT-ESG), 25.53 ± 1.01 (BTG)  - MBI: 52.99 ± 8.52 (BT-ESG), 54.92 ± 8.96 (BTG)  - Gender: 16/13 (BT-ESG), 17/13 (BTG)  - Paretic side: 15/14 (BT-ESG), 20/10 (BTG)  - Stroke type: 19/10 (BT-ESG), 18/12 (BTG) | - Modality: Balance training with weight shift-triggered electrical stimulation  - Dosage: Session length - 50 minutes; Frequency - 5 times per week; Total duration - 6 weeks | - Measurement tool: Postural assessment system (GB300; Metitur Ltd., Jyvaskyla, Finland)  - Sampling frequency: 50 Hz  - Measurement conditions: Eyes open and eyes closed | The comparator or control condition is the balance training group (BTG), which received balance training without weight shift-triggered electrical stimulation. | - Laboratory-based COP metrics: Postural sway  - Clinical balance scales: Berg Balance Scale (BBS), Timed Up and Go (TUG) test, functional reach test (FRT)  - Fall incidence or confidence questionnaires: Not mentioned | - Static balance: BT-ESG showed significant improvement in postural sway compared to BTG, with effect sizes of 0.88 (eyes-open) and 0.81 (eyes-closed).  - Dynamic balance: Significant improvements in TUG, BBS, and FRT in BT-ESG compared to BTG, with effect sizes of 0.90, 1.26, and 1.45, respectively.  - Lower-extremity motor function: Significant improvement in BT-ESG compared to BTG, with an effect size of 1.38.  - Activities of daily living: Significant improvement in BT-ESG compared to BTG, with an effect size of 2.04.  - Fall risk reduction: BBS improvement suggests mitigation of fall risk. |
| The effect of a motor relearning on balance and postural control in patients after stroke: An open-label randomized controlled trial  **Amer Ghrouz +6**  European Stroke Journal  2023 · | - Trial type: Randomized controlled trial (RCT) with parallel design  - Sample size: 63 subacute stroke patients (32 in MRP group, 31 in CPT group)  - Phase: Subacute  - Setting: Outpatient rehabilitation department | - Subacute stroke patients aged 18-85  - First-ever stroke within 1-6 months  - Hemiparesis with lower-limb muscle power of 2-4 on the MRC Muscle Scale  - Independent standing for at least 1 minute  - Ambulation of 25 feet/10 m (with or without assistive device)  - No major cognitive deficits (MoCA score > 20)  - No communication impairments  - No visual impairments  - No other related therapy or medical contraindications | - Modality: Task-specific training based on MRP for the intervention group; conventional physical therapy for the control group.  - Dosage: 8-week intervention, 3 sessions per week, 1 hour per session, totaling 24 sessions. | - Measurement tool: NedSVE/IBV ® system  - Platform: Dynamometric platform  - Parameters: Postural oscillation indicating COP displacement  - Sampling rate and force platform model: Not specified | Conventional physical therapy (CPT) | - Clinical balance scales: Berg Balance Scale (BBS)  - Laboratory-based COP metrics: Posturography (static posturography tests, limits of stability, rhythmic and directional control)  - Balance index global score | The MRP group showed significant improvements in balance and postural control, as evidenced by larger BBS and Balance Index scores, and enhanced postural control assessments, including LOS and rhythmic/directional control tests. These improvements are attributed to the MRP's dynamic and task-specific exercises, which may enhance motor learning and neural plasticity, potentially reducing fall risk. However, explicit links between COP changes and fall risk reduction are not directly mentioned. |

**1**
