# Supplementary material for: Modifying center of pressure to reduce fall risk in adult stroke survivors: a scoping review
Source: Front Neurol. 2026 Apr 23;17:1773299. doi: 10.3389/fneur.2026.1773299 (PMC13149131; doi:10.3389/fneur.2026.1773299)
Supplement: Supplementary File 2 — Stakeholder consultation: protocol, interview guides, and full thematic analysis (provided as a separate file; unchanged). [file Table_2.docx]

**Supplement S1 B. Extraction data, COP specific variables and Intervention Characterization**

| Paper | Force platform specifications (model, sampling frequency, calibration protocols) | COP parameters measured (sway area, path length, velocity, RMS, frequency measures) | Measurement conditions (eyes open/closed, surface type, stance width, trial duration) | Data processing methods (filtering specifications, artifact removal, normalization) | Quantification of change (absolute differences, percentage change, effect sizes, minimal detectable change) | Feedback modality (visual, auditory, haptic, multimodal) | Feedback timing (concurrent vs. terminal, delay specifications) | Target parameters (specific COP metrics being modified) | Progression criteria (how difficulty/challenge was advanced) | Dose specification (session frequency, duration, total intervention period) |
| --- | --- | --- | --- | --- | --- | --- | --- | --- | --- | --- |
| The effects of symmetric center of pressure displacement training with feedback on the gait of stroke patients  **Jin-Seop Kim**  Journal of Physical Therapy Science  2015 | Not mentioned (the paper does not provide specific details about the model, sampling frequency, or calibration protocols of the force platform) | Not mentioned (the paper does not provide information on COP parameters such as sway area, path length, velocity, RMS, or frequency measures) | - Eyes open: Yes  - Surface type: Not mentioned  - Stance width: Not mentioned  - Trial duration: 15 minutes per session | Not mentioned (the paper does not provide specific details on filtering specifications, artifact removal, or normalization methods) | - Step length: Experimental group pre-test mean = 31.8 cm, post-test mean = 44.2 cm; Control group pre-test mean = 30.2 cm, post-test mean = 33.0 cm  - Stride length: Experimental group pre-test mean = 62.0 cm, post-test mean = 87.3 cm; Control group pre-test mean = 65.3 cm, post-test mean = 64.7 cm  - Gait velocity: Experimental group pre-test mean = 0.7 m/s, post-test mean = 1.0 m/s; Control group pre-test mean = 0.8 m/s, post-test mean = 0.8 m/s  - Functional reach test: Experimental group pre-test mean = 18.6 cm, post-test mean = 26.2 cm; Control group pre-test mean = 17.4 cm, post-test mean = 23.0 cm | Visual | Concurrent feedback | COP displacement from the heel to the first metatarsal bone; lateral and antero/posterior COP displacement velocities | Not mentioned (the paper does not provide specific details on how difficulty or challenge was advanced during the training) | - Session frequency: Three times per week  - Duration: 15 minutes per session  - Total intervention period: Six weeks |
| Effects of Vibrotactile Biofeedback Providing Real-Time Pressure Information on Static Balance Ability and Weight Distribution Symmetry Index in Patients with Chronic Stroke  **Hongjun Kim +1**  Brain Science  2022 · | - Model: Wii balance board  - Sampling frequency: 100 Hz  - Calibration protocols: Not explicitly mentioned | - Sway Length: 91.11 cm (tactile biofeedback), 102.89 cm (visual biofeedback), 114.59 cm (no biofeedback)  - Sway Velocity: 3.06 cm/s (tactile biofeedback), 3.34 cm/s (visual biofeedback), 3.68 cm/s (no biofeedback) | - Eyes open: Yes  - Surface type: Wii balance board  - Stance width: Standard (both feet naturally positioned)  - Trial duration: 3 sets of 30 seconds each, with 3 minutes of rest between sets | - Sampling rate: 100 Hz  - Filtering: 10 Hz low-pass filter  - Artifact removal: Not mentioned  - Normalization: Not mentioned | - Sway Length: Tactile Biofeedback = 91.11 ± 19.27 cm, Visual Biofeedback = 102.89 ± 28.66 cm, No Biofeedback = 114.59 ± 28.78 cm  - Sway Velocity: Tactile Biofeedback = 3.06 ± 0.87 cm/s, Visual Biofeedback = 3.34 ± 1.10 cm/s, No Biofeedback = 3.68 ± 1.09 cm/s  - Weight Distribution Symmetry Index: Tactile Biofeedback = 10.85 ± 9.81%, Visual Biofeedback = 25.89 ± 17.65%, No Biofeedback = 39.21 ± 24.42% | - Vibrotactile (haptic)  - Visual  - No biofeedback | Concurrent feedback timing with real-time pressure information and immediate vibration stimulation. | - Sway length  - Sway velocity  - Weight distribution symmetry index | Not mentioned (the paper does not provide information on how difficulty or challenge was advanced) | - Session frequency: Each condition was applied once.  - Duration: Each condition was applied for 1 day.  - Total intervention period: 3 days (considering the 24-hour washout periods between conditions). |
| Identify the Alteration of Balance Control and Risk of Falling in Stroke Survivors During Obstacle Crossing Based on Kinematic Analysis  **Carmela Conte +6**  Frontiers in Neurology  2019 ·s | - Model: AMTI  - Dimensions: 464 mm × 508 mm × 83 mm  - Sampling frequency: 1 kHz  - Calibration protocols: Not mentioned | Not mentioned (the paper does not provide details on COP parameters such as sway area, path length, velocity, or frequency measures) | - Eyes: Not mentioned  - Surface type: Flat, smooth surface (implied by use of force plates and barefoot walking)  - Stance width: Not controlled or measured  - Trial duration: Variable, defined by crossing stride | - Data processing software: Vicon Nexus (Version 1.7.1)  - Kinematic data processing: Obtained during the crossing stride  - COM and COP calculation: Vicon Workstation software  - Normalization: RMS calculation for COM-COP distance  - Artifact removal: Not explicitly mentioned | - Absolute differences: Stroke survivors had smaller AP COM velocities and COM-COP distances compared to healthy controls.  - Percentage change: Not explicitly mentioned.  - Effect sizes: Correlations between COM velocity, COM-COP distance, and muscle strength indicate effect sizes, but specific values are not provided.  - Minimal detectable change: Not mentioned. | visual | Not mentioned (the paper does not discuss feedback timing or delay specifications) | - Distance between COM and COP in the AP direction during TLP and LLP  - Root mean square (RMS) of COM-COP distance during TLP and LLP | The progression criteria involved increasing the height of the obstacle to 10%, 20%, and 30% of the leg length, which challenged the balance control and motor strategies of stroke survivors. | Not mentioned (the paper does not provide details on session frequency, duration, or total intervention period) |
| Effects of Balance Exercise Assist Robot training for patients with hemiparetic stroke: a randomized controlled trial  **Seigo Inoue +5** | - Model: Kinetogravicorder G-7100  - Sampling frequency: Not mentioned  - Calibration protocols: Not mentioned | - Maximum COP movement, left-right, cm, mean (SD)  - Maximum COP movement, anterior-to-posterior, cm, mean (SD)  - Sway area, path length, velocity, RMS, frequency measures: Not mentioned | - Eyes: Open  - Surface type: Force plate  - Stance width: Shoulder-width apart  - Trial duration: Not explicitly mentioned | Not mentioned (the paper does not provide specific details on data processing methods such as filtering specifications, artifact removal, or normalization) | - Absolute differences: Post-intervention changes in Mini-BESTest scores were 3.5 (2.1) for BEAR, 3.4 (2.5) for IBT, and 1.2 (2.4) for CR. At follow-up, changes were 5.4 (2.8) for BEAR, 5.2 (3.1) for IBT, and 1.9 (2.5) for CR.  - Minimal clinically important difference (MCID): 4 points for the Mini-BESTest.  - Effect size: 1.025.  - Percentage change and minimal detectable change (MDC) are not explicitly mentioned. | Multimodal (visual and haptic) | Concurrent feedback; the robot adjusts difficulty levels based on real-time performance and provides augmented feedback during tasks. | - Maximum COP movement, left-right, cm  - Maximum COP movement, anterior-to-posterior, cm | The robot automatically changed the difficulty level of the balancing task (game) according to the level of achievement. | - Session frequency: 6 times a week  - Session duration: 18 minutes  - Total intervention period: 2 weeks |
| Balance Training With a Vibrotactile Biofeedback System Affects the Dynamical Structure of the Center of Pressure Trajectories in Chronic Stroke Patients  **Sonia Julia-Sanchez** +5  Frontiers in Human Neuroscience  2019 · | - Model: Wii Balance board  - Sampling frequency: 50 Hz  - Calibration protocols: Not mentioned | - Sway area: No significant change  - Path length: No significant change  - Velocity: No significant change  - RMS: Not mentioned  - Frequency measures: Not mentioned | - Eyes: Open  - Surface type: Rubber foam mat  - Stance width: Not mentioned  - Trial duration: 30 seconds | - Filtering: Savitzky-Golay filter (order 3, length 7)  - Artifact removal: Not explicitly mentioned  - Normalization: Not explicitly mentioned | - ML direction: Absolute difference = 0.09, Percentage change = 22.5%  - AP direction: No significant change | Haptic | Concurrent feedback; no specific delay specifications mentioned | - Slow-scale dynamics of ML CoP: Decrease in scaling exponent from pre-training to post-training (α = 0.40 ± 0.13 vs. 0.31 ± 0.09), indicating stronger anti-persistent dynamics.  - Error correction strategy in ML CoP: Change in slow-scale scaling exponent suggesting tighter control over body sway. | - Participants underwent 45 minutes of BF training twice a week for 2 weeks.  - Training consisted of two task-oriented balance exercises: standing on a rubber foam mat and weight-shifting to the paralyzed limb.  - Each session included 10 repetitions of the balance task with short intervals.  - The BF threshold setting was reset daily, with a target area defined as 90% of the pre-measured 95% confidence circle area. | - Session frequency: Twice a week  - Duration: 45 minutes per session  - Total intervention period: 4 weeks |
| Validity and reliability of center of pressure measures to quantify trunk control ability in individuals after stroke in subacute phase during unstable sitting test  **Anne-Violette Bruyneel +6**  Heliyon  2022 · | - Model: kin etools 2015, Kicarre company  - Dimensions: 60 cm Â 45 cm x 6.2 cm  - Force transducers: SP4C3-MR, precision C3, HBM Â  - Sampling frequency: 100 Hz  - Calibration protocols: Not explicitly mentioned, but signals were filtered with a 5 th Order Butterworth Low Pass Filter (cut-off set to 45 Hz) | - Sway area: Ellipse area (CI95%)  - Path length: Length (total, anteroposterior, and mediolateral)  - Velocity: Mean and maximum velocity  - RMS: Not mentioned  - Frequency measures: Not mentioned | - Eyes: Open  - Surface type: Seesaw  - Stance width: Feet flat on the floor (implied standard stance width)  - Trial duration: 10 seconds | - Filtering specifications: 5th Order Butterworth Low Pass Filter with a cut-off set to 45 Hz.  - Sampling frequency: 100 Hz.  - Artifact removal and normalization: Not mentioned. | - Minimum Detectable Change (MDC): Provided in tables for CoP parameters.  - Standard Error of Measurement (SEM): Provided in tables for CoP parameters.  - Absolute differences and percentage change: Not explicitly mentioned, but can be inferred from SEM and MDC values.  - Effect sizes: Not explicitly mentioned, but ICC values can provide insight into effect magnitude. | Not mentioned (the paper does not specify the feedback modality) | Concurrent feedback; no specific delay specifications mentioned. | - Total path length  - Anteroposterior length  - Mediolateral length  - Ellipse area  - Deltas (anteroposterior and mediolateral)  - Mean velocity  - Maximum velocity  - Variability | The progression criteria involved changing the direction of instability using a seesaw to induce disturbances in either the mediolateral or anteroposterior direction, and analyzing various CoP parameters (length, ellipse area, deltas, mean and maximum velocity, variability) to assess how difficulty or challenge was advanced. | - Session frequency: 2 sessions per participant  - Duration: 10 seconds per test  - Total intervention period: Less than a day (single day of testing) |
| Different weight shift trainings can improve the balance performance of patients with a chronic stroke A randomized controlled trial  **Wan-Chun Liao** +4  Medicine  2018 · | Not mentioned (the paper does not provide specific details about the force platform specifications) | Not mentioned (the paper does not provide information on COP parameters such as sway area, path length, velocity, RMS, or frequency measures) | Not mentioned (the paper does not provide specific measurement conditions for the balance CAT or TUG tests) | - Data analysis software: SPSS 14.0  - Statistical methods: One-way ANOVA, Chi-squared test, repeated measures ANOVA, intention-to-treat analysis with last observation carried forward  - Effect size calculations: Partial eta-square (h 2 ), Cohen d  - No specific mention of filtering specifications, artifact removal, or normalization | - Effect sizes for balance CAT:  - BT group: 1.40 (week 6), 1.37 (week 10), 1.15 (week 18)  - LW group: 0.96 (week 6), 1.28 (week 10), 1.10 (week 18)  - Control group: 0.22 (week 6), 0.13 (week 10), 0.22 (week 18)  - Absolute differences in balance CAT scores:  - BT group: Increased from 7.41 to 8.24 points  - LW group: Increased from 7.49 to 8.33 points  - TUG test: Decrease in time taken to complete the test, indicating improved dynamic shift ability | visual | Not mentioned (the paper does not specify the feedback timing or delay specifications) | Not mentioned (the paper does not explicitly mention specific COP metrics being modified) | Not mentioned (the paper does not explicitly describe a progression criteria for advancing difficulty or challenge in the training) | - Session frequency: 3 days per week  - Session duration: 20 minutes per session  - Total intervention period: 6 weeks |
| Effects of Real-Time Feedback Methods on Static Balance Training in Stroke Patients: A Randomized Controlled Trial  **Il-Ho Kwon +3**  Healthcare  2024 · | - Model: BP400600, AMTI, Waterton, MA, USA  - Dimensions: 400 mm × 600 mm  - Sampling frequency: Not mentioned  - Calibration protocols: Not mentioned | - Sway area: Area 95% (cm²)  - Path length: Sway length (cm)  - Velocity: Sway velocity (cm/s)  - RMS: Not mentioned  - Frequency measures: Not mentioned | - Eyes: Open and Closed  - Surface Type: Force Plate  - Stance Width: Not explicitly mentioned (implied comfortable standing position)  - Trial Duration: 1 minute | - Data processing software: BioAnalysis Version 2.2  - Statistical analysis software: IBM SPSS Statistics version 25.0  - Normality test: Shapiro-Wilk test  - Statistical method: Two-way ANOVA with repeated measures | - Absolute differences: Sway length (KR: 387.28 cm to 320.33 cm; KP: 402.71 cm to 367.78 cm), Sway velocity (KR: 6.35 cm/s to 5.97 cm/s; KP: 6.73 cm/s to 6.33 cm/s), Area 95% (KR: 4.10 cm² to 2.92 cm²; KP: 3.38 cm² to 2.67 cm²)  - Percentage change: Sway length (KR: -17.3%; KP: -8.7%), Sway velocity (KR: -6.1%; KP: -5.9%), Area 95% (KR: -28.8%; KP: -21.1%)  - Effect sizes: Significant differences for group × time interaction in sway length and area 95% (p < 0.05)  - Minimal detectable change: Not explicitly mentioned | Visual | Concurrent, no delay specifications mentioned | - Sway length (cm; total length in all directions of the COP)  - Sway velocity (cm/s; average velocity of the COP)  - Area 95% (cm²; 95% of the total area moved by the COP) | Not mentioned (the paper does not provide specific details on how the difficulty or challenge was advanced or progressed during the training sessions) | - Session frequency: Five times a week  - Duration: 30 minutes per session  - Total intervention period: Four weeks |
| Balance Training with Weight Shift-Triggered Electrical Stimulation for Stroke Patients: A Randomized Controlled Trial  **Kyeongjin Lee**  Brain Science  2023 · | - Model: GB300; Metitur Ltd., Jyvaskyla, Finland  - Sampling frequency: 50 Hz  - Calibration protocols: Not mentioned | - Medial and lateral sway speed  - Anterior and posterior sway speed  - Velocity of moment | - Eyes open/closed: Yes, measurements were taken with both eyes open and closed.  - Surface type: Not explicitly mentioned, but implied to be a movable triangular platform.  - Stance width: Not mentioned.  - Trial duration: 30 seconds for each trial, repeated three times. | - Data was averaged from triplicate evaluations by three physiotherapists.  - Shapiro-Wilk test was used to confirm data normality.  - Two-way repeated measure ANOVA was used for statistical analysis.  - Effect sizes and 95% confidence intervals were calculated.  - Statistical significance level (α) was set at p < 0.05. | - Effect sizes:  - Static balance (eyes-open): 0.88; 95% CI: -1.16 to -1.30  - Static balance (eyes-closed): 0.81; 95% CI: -1.22 to -0.27  - Dynamic balance (TUG): 0.90; 95% CI: -4.67 to -1.25  - Dynamic balance (BBS): 1.26; 95% CI: -2.84 to 6.83  - Dynamic balance (FRT): 1.45; 95% CI: 1.92 to 4.08  - Lower-extremity motor function: 1.38; 95% CI: 2.25 to 4.97  - Activities of daily living: 2.04; 95% CI: 2.04 to 937  - Percentage changes:  - Postural sway improvements:  - Eyes-open: mediolateral speed increase of 28.2%, anterioposterior speed of 21.5%, velocity moment of 34.4%  - Eyes-closed: mediolateral speed increase of 26.2%, anterioposterior speed of 15.2%, velocity moment of 26.5%  - TUG time decrease: 14%  - FRT improvement: 23.6%  - BBS improvement: 21.5%  - Fugl-Meyer assessment improvement: 31.4% | Haptic | Concurrent feedback; triggered when 90% of the measured value is reached on the unaffected side, with real-time adjustment based on individual characteristics. | - Static balance: Velocity moment, medial and lateral sway speed, anterior and posterior sway speed  - Dynamic balance: TUG test, Berg Balance Scale (BBS), functional reach test (FRT)  - Lower-extremity motor function: Fugl-Meyer assessment lower-extremity scale  - Activities of daily living: Modified Barthel Index (MBI) | - Participants were selected based on specific inclusion criteria (e.g., understanding verbal instructions, Mini-Mental State Exam score ≥24, ability to stand independently).  - Training was conducted 5 times a week for 6 weeks, with each session lasting 50 minutes.  - Progression was evaluated using pre- and post-tests to assess changes in balance and motor function.  - Statistical analysis included two-way repeated measure ANOVA and effect size calculations to assess training effects.  - Participants had to complete at least 80% of the program to be included in the final analysis. | - Session frequency: 5 times per week  - Session duration: 50 minutes per session  - Total intervention period: 6 weeks |
| The effect of a motor relearning on balance and postural control in patients after stroke: An open-label randomized controlled trial  **Amer Ghrouz** +6  European Stroke Journal  2023 · | Not mentioned (the paper does not provide specific details about the force platform model, sampling frequency, or calibration protocols) | Not mentioned (the paper does not specify the COP parameters such as sway area, path length, velocity, RMS, or frequency measures) | - Eyes open/closed: Yes, tests were conducted with eyes open (ROA) and eyes closed (ROC).  - Surface type: Firm surface and foam cushion.  - Stance width: Not mentioned.  - Trial duration: 30 seconds for Romberg tests. | Not mentioned (the paper does not explicitly detail data processing methods such as filtering specifications, artifact removal, or normalization) | - Effect sizes: d=2.98 for BBS, d=2.83 for Balance Index  - Absolute differences: Mean BBS score change - MRP: 13.78, CPT: 6.90  - Minimal detectable change: 6 BBS points | Not mentioned (the paper does not specify the feedback modality used in the MRP or CPT interventions) | Not mentioned (the paper does not specify the feedback timing or delay specifications) | Not mentioned (the paper does not explicitly mention specific COP metrics being modified) | The progression criteria in the Motor Relearning Program (MRP) involve gradually increasing difficulty with feedback, incorporating more dynamic and complex balance tasks, and using practice variability, specificity, and feedback to enhance motor skill learning and retention. | - Session frequency: 3 sessions per week  - Session duration: 1 hour per session  - Total intervention period: 8 weeks |
