# Supplementary material for: Modifying center of pressure to reduce fall risk in adult stroke survivors: a scoping review
Source: Front Neurol. 2026 Apr 23;17:1773299. doi: 10.3389/fneur.2026.1773299 (PMC13149131; doi:10.3389/fneur.2026.1773299)
Supplement: Supplementary File 3 — Combined Appendix—Standardization framework & minimum COP protocol (Section A); Economic analysis & cost-effectiveness templates (Section B); Research agenda & methodological recommendations (Section C); Supplementary Table S1:Force-platform systems and measurement specifications (Section D). [file Table_3.docx]

# **S2 Supplementary Appendix: Stakeholder Consultation Findings**

## **Translating Center of Pressure Interventions into Clinical Practice: A Multidisciplinary Stakeholder Consultation**

### **Abstract**

To enhance the clinical relevance and translational potential of our scoping review on center of pressure (COP) interventions for fall prevention in stroke survivors, we conducted structured consultations with a multidisciplinary stakeholder panel (n=7). Through iterative thematic analysis of two 90-minute focus groups, we identified critical insights regarding COP parameter interpretability, intervention feasibility, outcome priorities, and implementation barriers. Stroke survivors emphasized the importance of visual feedback and functional relevance, while clinicians highlighted standardization needs and integration challenges. Technology experts identified interface simplification as paramount for clinical adoption. This supplementary analysis provides essential context for translating laboratory-based COP research into routine neurorehabilitation practice.

### **Introduction**

The translation of biomechanical research into clinical practice requires careful consideration of multiple stakeholder perspectives. While our primary review identified promising COP-based interventions for post-stroke fall prevention, questions remained regarding real-world implementation feasibility and clinical interpretability. This stakeholder consultation aimed to bridge the research-practice gap by systematically capturing insights from those most directly affected by these interventions.

### **Methods**

We convened a purposively sampled stakeholder panel representing key perspectives in stroke rehabilitation. The panel comprised three stroke survivors (mean age 58 years, range 6-36 months post-stroke, varying functional levels), two senior neurological physiotherapists (mean experience 13.5 years), one clinical scientist specializing in balance assessment (PhD biomechanics, 10 years clinical research), and one biomedical engineer with force platform expertise (MSc, 8 years industry experience).

Two structured focus groups were conducted using a hybrid format to accommodate participant preferences and mobility constraints. Sessions were audio-recorded, transcribed verbatim, and analyzed using framework analysis guided by implementation science principles. The semi-structured interview guide explored four domains: COP parameter interpretability, intervention feasibility, outcome priorities, and implementation factors.

**Table S1** presents the detailed characteristics of our purposively sampled stakeholder panel. The diversity of perspectives—from recent to chronic stroke experiences, acute to community settings, and research to clinical applications—ensured comprehensive coverage of implementation considerations.

**Table S1. Stakeholder Panel Characteristics and Representation**

| **Stakeholder Category** | **Identifier** | **Relevant Characteristics** | **Unique Perspective Contributed** |
| --- | --- | --- | --- |
| **Stroke Survivors**  (n = 3) | Participant A | 58y, 18mo post-stroke, independent ambulation, mild balance impairment, returned to part-time work | Technology adoption in functional recovery, workplace reintegration concerns |
|  | Participant B | 71y, 36mo post-stroke, walking aid user, fall history (3 falls in past year), lives alone | Fall fear impact, safety concerns, aging with stroke-related disability |
|  | Participant C | 45y, 8mo post-stroke, left hemiparesis, young family, high motivation | Family impact, return to premorbid activities, long-term recovery goals |
| **Clinical Physiotherapists**  (n = 2) | PT1 | Senior neurological specialist, 15y experience, acute stroke unit, MSc Neurological Physiotherapy | Acute implementation challenges, multidisciplinary team integration |
|  | PT2 | Community specialist, 12y experience, outpatient neurorehabilitation, certified in balance assessment | Community translation, resource limitations, long-term management |
| **Clinical Neuro Scientist**  (n = 1) | CS | MSc Biomechanics, Clinical Neuroscience, 10y clinical research.Clinical experience with force plates | Measurement standardization, research-practice gap, evidence quality |
| **Biomedical Engineer**  (n = 1) | BE | MSc Biomedical Engineering, 3y force platform development, | Technology adaptation, cost-effectiveness, user interface design |

### **Findings**

**Table S2. Thematic Analysis of Stakeholder Perspectives on COP Intervention Implementation**

| **Theme** | **Subtheme** | **Representative Quote** | **Stakeholder Source** |
| --- | --- | --- | --- |
| **Understanding and Engagement** | Initial conceptual barriers | "Center of pressure meant nothing to me until I saw it on screen" | Participant A |
|  | Visual feedback preference | "Numbers don't help, but seeing the dot move when I lean—that's real" | Participant B |
|  | Motivational elements | "The games made me want to beat my score from yesterday" | Participant C |
|  | Clinical explanation challenges | "I can explain sway speed easily, but frequency domains? Forget it in a busy clinic" | PT1 |
| *Practical Implementation Concerns* | | | |
| **Barriers and Facilitators** | Equipment intimidation | "That big platform looked like something from a hospital, not therapy" | Participant B |
|  | Time constraints | "Adding 15 minutes means another patient doesn't get seen" | PT2 |
|  | Documentation value | "Objective data transforms funding conversations completely" | PT1 |
|  | Technology mismatch | "We're using Formula 1 cars when clinics need reliable sedans" | BE |
|  | Standardization urgency | "Without standards, a 30% improvement is meaningless across sites" | CS |
| *Outcome Priorities and Success Metrics* | | | |
| **Meaningful Outcomes** | Functional relevance | "Show me I can reach my grandchild's toys without falling" | Participant C |
|  | Confidence restoration | "Less fear when walking to the shops matters more than any number" | Participant A |
|  | Clinical utility | "Sway velocity correlates with function—that's what I need" | PT2 |
|  | Research translation | "Lab measures must map to real-world fall risk" | CS |

**Table S2** presents the complete thematic analysis, organizing stakeholder insights into major themes and subthemes. The following sections explore these themes in detail, beginning with the patient perspective.

#### **The Patient Perspective: From Abstract Metrics to Embodied Understanding**

Stroke survivors consistently expressed initial confusion regarding COP concepts, which transformed into engagement once visual representations were provided. One participant articulated this transformation: *"When I first heard 'center of pressure,' it meant nothing. But as I shifted my weight, I saw that dot move on the screen, and suddenly I understood why I kept falling to the right. It was like seeing inside my own balance."* (see Table S2 for complete thematic analysis)

This embodied understanding proved crucial for intervention engagement. Participants unanimously preferred real-time visual feedback over numerical displays, with gaming elements particularly motivating for younger survivors. However, technology-related anxiety emerged as a significant concern, especially among older participants who found large force platforms "intimidating" and "overly medical."

Fear of falling paradoxically increased during initial platform assessments for two participants, highlighting the need for careful environmental setup and psychological support. Session duration also proved challenging, with fatigue limiting engagement beyond 30 minutes for those with moderate impairments. Participants strongly advocated for clear, week-by-week progress visualization using functional rather than biomechanical metrics.

#### **Clinical Integration: Balancing Innovation with Pragmatism**

Physiotherapists demonstrated enthusiasm for objective balance measurement while expressing pragmatic concerns about implementation. Both therapists independently identified sway velocity as the most clinically intuitive parameter, with one noting: *"I can explain sway speed to patients and families easily—faster sway means less stable. But try explaining fractal dynamics or frequency domain measures in a busy clinic."*

Integration challenges centered on time constraints, with COP assessment adding 10-15 minutes to standard sessions. However, therapists recognized the value of documentation, particularly for insurance justification and progress monitoring. One therapist observed, *"Having objective data transforms conversations with funders. Instead of saying 'balance improved,' I can show percentage changes in weight symmetry and sway reduction."*

Both therapists emphasized the need for flexible protocols accommodating cognitive impairment and varying fatigue levels. They proposed a phased implementation approach: beginning with assessment only, gradually introducing feedback as patients developed platform comfort and conceptual understanding. Successful integration required embedding COP measures within, rather than adding to, existing therapeutic activities.

#### **Technical Perspectives: Bridging the Translation Gap**

The clinical scientist provided critical insights into measurement standardization, emphasizing that methodological heterogeneity undermines clinical translation: *"Without standardized protocols, we cannot develop meaningful clinical thresholds or normative databases. A patient's 30% improvement means nothing without context."*

Specific standardization recommendations emerged through interdisciplinary discussion: minimum 50 Hz sampling frequency, 30-second trial duration, three trials per condition, and consistent 10 Hz low-pass Butterworth filtering. The scientist advocated for a minimal clinical dataset comprising sway path length, mean velocity (anteroposterior and mediolateral), 95% confidence ellipse area, and weight-bearing symmetry index. The clinical scientist's specific standardization recommendations, endorsed by all technical stakeholders, are summarized in Table S3.

**Table S3. Consensus Recommendations for COP Parameter Selection and Clinical Implementation**

| **COP Parameter** | **Clinical Interpretability^a^** | **Patient Understanding^b^** | **Implementation Feasibility^c^** | **Priority Ranking^d^** |
| --- | --- | --- | --- | --- |
| **Sway velocity (cm/s)** | **High (7/7)** | **High (3/3)** | **High (4/4)** | **1** |
| **Weight-bearing symmetry (%)** | **High (7/7)** | **High (3/3)** | **High (4/4)** | **2** |
| **Limits of stability (cm²)** | **High (6/7)** | **Moderate (2/3)** | **High (4/4)** | **3** |
| **95% confidence ellipse area (cm²)** | **Moderate (5/7)** | **Low (1/3)** | **High (4/4)** | **4** |
| **Total sway path length (cm)** | **Moderate (5/7)** | **Moderate (2/3)** | **High (4/4)** | **5** |
| **Frequency domain measures** | **Low (2/7)** | **Low (0/3)** | **Moderate (2/4)** | **6** |
| **Fractal dynamics (DFA)** | **Low (1/7)** | **Low (0/3)** | **Low (1/4)** | **7** |

**^a^Rated by all stakeholders (n=7) as high/moderate/low with consensus numbers shown**

**^b^Rated by stroke survivors only (n=3)**

**^c^Rated by clinical stakeholders (n=4: PT1, PT2, CS, BE)**

**^d^Final priority ranking based on combined scores across all domains**

**DFA, detrended fluctuation analysis**

The biomedical engineer identified a fundamental mismatch between research-oriented systems and clinical needs: *"Current platforms are Formula 1 cars when clinics need reliable sedans. We must simplify interfaces without sacrificing measurement validity."* Key technological barriers included poor electronic health record integration, complex user interfaces designed for researchers rather than clinicians, and the absence of automated interpretation algorithms.

#### **Convergent Themes: Toward Implementation**

Despite diverse perspectives, several convergent themes emerged. All stakeholders agreed that visual feedback enhanced engagement and understanding, though optimal presentation varied by cognitive capacity. The importance of linking abstract COP metrics to functional outcomes resonated across groups, with fall prevention and activity participation prioritized over biomechanical precision.

Despite diverse backgrounds, stakeholders demonstrated remarkable consensus regarding priority COP parameters for clinical implementation (**Table S3**). Sway velocity and weight-bearing symmetry emerged as universally endorsed measures, combining clinical interpretability with patient understanding.

Cost emerged as a universal concern, though perspectives differed. Stroke survivors are worried about treatment accessibility, clinicians are concerned about departmental budgets, and technology experts are worried about market viability. Creative solutions proposed included tiered technology options, equipment sharing between facilities, and advocacy for insurance coverage based on fall prevention cost-effectiveness.

Training needs extended beyond technical operation to include patient communication strategies, safety protocols, and clinical reasoning for COP-based treatment planning. Stakeholders recommended developing standardized competencies with mentorship programs supporting skill development.

### **Synthesis and Recommendations**

This consultation revealed both enthusiasm for COP interventions and legitimate implementation concerns requiring systematic addressing. **Table S4** presents the stakeholder-informed implementation roadmap, organizing recommendations by timeline and assigning clear accountability. This structured approach emerged through iterative discussion and reflects practical considerations raised throughout the consultation.

**Table S4. Implementation Roadmap: Stakeholder-Informed Priorities and Timelines**

| **Timeframe** | **Priority Action** | **Specific Deliverables** | **Lead Stakeholder** | **Success Metrics** |
| --- | --- | --- | --- | --- |
| **Immediate (0-12 months)** | | | | |
| **Immediate** | **Measurement standardization** | **• Consensus protocol document**  **• Minimum dataset specification**  **• Calibration procedures** | **Clinical scientists** | **≥80% adoption in trials** |
|  | **Patient education materials** | **• Visual explanation tools**  **• Plain language guides**  **• Progress tracking templates** | **Stroke survivors + PTs** | **Patient comprehension >90%** |
|  | **Pilot implementation** | **• 3-site feasibility study**  **• Process evaluation**  **• Cost analysis** | **Clinical teams** | **Recruitment >75% target** |
| **Short-term (1-2 years)** | | | | |
| **Short-term** | **Technology simplification** | **• Clinical user interface**  **• Automated reporting**  **• EHR integration** | **Engineers + PTs** | **Setup time <10 min** |
|  | **Effectiveness trials** | **• Multicenter RCT**  **• Fall outcome tracking**  **• PROM development** | **Research teams** | **n>200, 80% retention** |
|  | **Training program** | **• Competency framework**  **• Online modules**  **• Mentorship network** | **Professional bodies** | **500+ trained clinicians** |
| **Long-term (2-5 years)** | | | | |
| **Long-term** | **Guideline integration** | **• Clinical practice guidelines**  **• Quality indicators**  **• Audit tools** | **Professional societies** | **Inclusion in ≥3 guidelines** |
|  | **Coverage advocacy** | **• Economic evaluation**  **• Policy briefs**  **• Payer engagement** | **All stakeholders** | **Insurance coverage in ≥50% markets** |
|  | **AI personalization** | **• Predictive algorithms**  **• Adaptive protocols**  **• Decision support tools** | **Tech + clinical teams** | **Algorithm accuracy >85%** |

The following recommendations emerged through stakeholder consensus:

**Immediate Priorities (0-12 months):** Development of consensus measurement standards through professional society engagement; creation of patient education materials using stakeholder-validated language; pilot implementation studies in diverse clinical settings with embedded process evaluation.

**Short-term Goals (1-2 years):** Multicenter effectiveness trials incorporating patient-reported outcomes and fall incidence; technology simplification initiatives focusing on clinical user interfaces; development of clinical decision algorithms for patient selection and progression.

**Long-term Vision (2-5 years):** Integration into clinical practice guidelines with clear indications and protocols; establishment of normative databases stratified by age, chronicity, and severity; insurance coverage based on demonstrated cost-effectiveness; artificial intelligence applications for personalized treatment protocols.

### **Clinical Implications**

The stakeholder consultation fundamentally shaped our understanding of COP intervention translation. Patient perspectives highlighted the critical importance of meaningful feedback presentation and functional goal alignment. Clinical insights emphasized pragmatic integration within existing workflows rather than standalone interventions. Technical expertise identified standardization and simplification as prerequisites for widespread adoption.

Most significantly, the consultation revealed that successful implementation requires coordinated effort across multiple domains: technology development, clinical training, patient education, and healthcare policy. Single-domain solutions will likely fail without considering the complex ecosystem of stroke rehabilitation.

### **Implementation Barriers and Solutions**

The consultation identified multiple implementation barriers spanning economic, technical, clinical, and psychosocial domains. **Table S5** presents these challenges alongside stakeholder-proposed solutions, providing a practical framework for addressing implementation obstacles. Notably, stakeholders emphasized that isolated solutions would likely fail; successful implementation requires coordinated efforts across all barrier categories.

**Table S5. Barriers to Implementation and Stakeholder-Proposed Solutions**

| **Barrier Category** | **Specific Challenge** | **Stakeholder-Proposed Solution** | **Implementation Requirements** |
| --- | --- | --- | --- |
| **Economic** | **High equipment costs ($5,000-50,000)** | **• Tiered technology options**  **• Equipment sharing models**  **• Leasing programs** | **• Business case development**  **• Regional coordination**  **• Vendor partnerships** |
|  | **Limited reimbursement** | **• Evidence-based coverage advocacy**  **• Bundled payment integration**  **• Outcome-based contracts** | **• Cost-effectiveness data**  **• Payer engagement**  **• Quality metrics** |
|  | **Staff training costs** | **• Online training modules**  **• Train-the-trainer models**  **• Competency-based certification** | **• Curriculum development**  **• Protected education time**  **• Ongoing support** |
| **Technical** | **Complex user interfaces** | **• Simplified clinical dashboards**  **• One-touch operation**  **• Visual-first design** | **• User experience testing**  **• Iterative refinement**  **• Clinical input** |
|  | **Poor EHR integration** | **• Standardized data formats**  **• API development**  **• Automated transfer protocols** | **• IT collaboration**  **• Interoperability standards**  **• Security compliance** |
|  | **Interpretation complexity** | **• Clinical decision algorithms**  **• Traffic light systems**  **• Automated recommendations** | **• Algorithm validation**  **• Clinical testing**  **• Regular updates** |
| **Clinical** | **Time constraints** | **• Integrated assessment protocols**  **• Concurrent interventions**  **• Efficient workflows** | **• Workflow analysis**  **• Protocol optimization**  **• Staff scheduling** |
|  | **Space limitations** | **• Portable systems**  **• Multi-use spaces**  **• Mobile units** | **• Equipment specifications**  **• Safety protocols**  **• Storage solutions** |
|  | **Patient heterogeneity** | **• Flexible protocols**  **• Cognitive adaptations**  **• Severity stratification** | **• Protocol variants**  **• Training materials**  **• Clinical pathways** |
| **Psychosocial** | **Technology anxiety** | **• Gradual introduction**  **• Peer mentoring**  **• Success stories** | **• Patient ambassadors**  **• Support groups**  **• Family engagement** |
|  | **Fall fear paradox** | **• Environmental optimization**  **• Psychological support**  **• Graded exposure** | **• Safety equipment**  **• Staff training**  **• Protocol modification** |
|  | **Motivation maintenance** | **• Gamification elements**  **• Personal goal setting**  **• Progress visualization** | **• Software features**  **• Goal-setting tools**  **• Feedback systems** |

### **Limitations**

This consultation involved stakeholders from a single healthcare system, potentially limiting generalizability. The small sample size, while appropriate for qualitative inquiry, may not capture all relevant perspectives. Notably absent were perspectives from healthcare administrators, insurance representatives, and family caregivers—all crucial for implementation success. Future consultations should expand stakeholder representation and explore cross-cultural implementation factors.

### **Conclusions**

Stakeholder consultation transformed our scoping review from an academic exercise into a translational roadmap. By centering patient experiences, clinical realities, and technical possibilities, we identified both the promise and prerequisites for COP intervention implementation. The path forward requires abandoning purely biomechanical perspectives in favor of integrated, patient-centered approaches that honor the complexity of stroke rehabilitation.

The unanimous stakeholder support for COP interventions, tempered by pragmatic implementation concerns, suggests a field poised for clinical translation pending systematic address of identified barriers. Success will require unprecedented collaboration between researchers, clinicians, technology developers, and—most importantly—stroke survivors themselves. Only through such collaboration can we transform promising laboratory findings into meaningful clinical improvements.

### **Acknowledgments**

We gratefully acknowledge the stroke survivors who shared their experiences and insights, contributing invaluable perspectives often absent from biomechanical research. We thank the clinical staff who participated despite busy schedules and the technology experts who bridged engineering and clinical domains. This consultation exemplifies the collaborative spirit necessary for advancing stroke rehabilitation.

### **Funding**

Stakeholder consultation activities were voluntary and not funded.

### **Competing Interests**

The biomedical engineer disclosed consulting relationships with force platform manufacturers, though these did not influence the consultation process or findings. Other stakeholders declared no competing interests.

### **Data Availability**

Anonymized focus group transcripts and thematic analysis frameworks are available upon reasonable request, subject to participant consent and institutional ethics approval.
