# Supplementary material for: Modifying center of pressure to reduce fall risk in adult stroke survivors: a scoping review
Source: Front Neurol. 2026 Apr 23;17:1773299. doi: 10.3389/fneur.2026.1773299 (PMC13149131; doi:10.3389/fneur.2026.1773299)
Supplement: Supplementary File 4 — Full database search strategies (terms, operators, and limits) for PubMed, MEDLINE, and the Cochrane Library (search executed 1 June 2025). [file Table_4.docx]

**Supplementary File S3. Combined Appendix — Methods & Implementation Pack**

This combined appendix consolidates materials previously divided among several files and figures: A) Standardization framework and minimum COP protocol (checklist); B) Templates for economic analysis and cost-effectiveness projection; C) Detailed research agenda and methodological suggestions; D) S‑Table S1 — Force‑platform systems and measurement specifications. These sections are cited in the manuscript as “Supplementary File S3 (Combined Appendix)”.

**Contents**

Section A. Standardization framework & minimum COP protocol (checklist)

Section B. Economic analysis & cost‑effectiveness projections (templates)

Section C. Research agenda & methodological recommendations

Section D. S‑Table S1 — Force‑platform systems and measurement specifications

**Section A. Standardization framework & minimum COP protocol (checklist)**

- Sampling frequency: ≥50 Hz (captures ≥99% of quiet‑stance sway spectrum).
- Trial duration: 30 s per condition; 3 trials (reliability vs. fatigue balance).
- Stance: Feet parallel; medial borders at ~10% body height; arms by side; barefoot or standardized footwear.
- Environment: Document visual condition (EO/EC), surface, ambient noise, time of day, medications affecting balance.
- Processing: 10 Hz low‑pass Butterworth (4th–5th order); report filters/software; handle outliers consistently.
- Core measures: Mean COP velocity (AP, ML), 95% ellipse area, weight‑bearing symmetry index; report units.
- Reporting: Provide raw means, SD, and change (absolute and %); include MDC/SEM when available.

| Checklist Item | Yes/No | Notes / Values |
| --- | --- | --- |
| Sampling frequency ≥50 Hz documented | □ Yes □ No |  |
| Trial duration 30 s, 3 trials per condition | □ Yes □ No |  |
| Stance standardization recorded | □ Yes □ No |  |
| Visual condition (EO/EC) and surface recorded | □ Yes □ No |  |
| Filter type & cut‑off reported | □ Yes □ No |  |
| Core measures exported (velocity AP/ML, ellipse area, symmetry) | □ Yes □ No |  |
| Environment and medications documented | □ Yes □ No |  |
| MDC/SEM referenced (or stated as NR) | □ Yes □ No |  |
| Adverse events and patient tolerance recorded | □ Yes □ No |  |

**Section B. Economic analysis & cost‑effectiveness projections (templates)**

| Assumption | Symbol | Default / Local Value |
| --- | --- | --- |
| Baseline 6‑month fall probability (chronic stroke) | p0 | e.g., 0.45 |
| Effect per 0.5 cm/s COP sway velocity reduction | Δp | e.g., −0.18 (−18% rel. change) |
| Expected sway velocity change from intervention | Δv | site‑specific |
| Average cost per injurious fall (payer perspective) | C_fall | e.g., $20,000 |
| Equipment/training amortized cost per patient | C_impl | site‑specific |
| Program delivery cost per patient | C_prog | site‑specific |

Key formulae: p1 = p0 × (1 + Δp × (Δv / 0.5)); ΔFalls = p0 − p1; Benefit = ΔFalls × C_fall; Net = Benefit − (C_impl + C_prog); BCR = Benefit / (C_impl + C_prog)

| Scenario | Δv (cm/s) | p1 (calc) | Benefit ($) | Net ($) |
| --- | --- | --- | --- | --- |
| Conservative |  |  |  |  |
| Base case |  |  |  |  |
| Optimistic |  |  |  |  |

Note: Replace defaults with your local costs and rates.

**Section C. Research agenda & methodological recommendations**

| Horizon | Priority Studies | Design / Notes |
| --- | --- | --- |
| Immediate (0–12 mo) | Consensus protocol; Cross‑platform validation; Clinical prediction rule | Delphi + lab/clinic paired recordings; Retrospective cohort |
| Short‑term (12–24 mo) | Dose–response multicenter RCT; Pragmatic effectiveness trial; Economic evaluation | Factorial/adaptive design; Registry‑embedded trial; Payer & societal perspectives |
| Long‑term (24+ mo) | Precision rehab algorithms; Learning health‑system; Guideline integration | Repeated COP+clinical outcomes; continuous QI cycles |

**Section D. S‑Table S1 — Force‑platform systems and measurement specifications**

S‑Table S1 (FINAL). Populated from the Week‑14 extraction for the nine included studies. ‘NR’ indicates not reported in the source article.

| Study (Author, Year) | Platform Model | Sampling (Hz) | Calibration | Software | COP Metrics | Filter (cut‑off) | Trial Duration | Stance / EO–EC |
| --- | --- | --- | --- | --- | --- | --- | --- | --- |
| Kim 2015 | NR (biofeedback plate unspecified); GAITRite® walkway for gait outcomes | NR | NR | NR | Targeted COP displacement/velocities (AP/ML) reported narratively; COP details NR | NR | Training: 15 min/session (6 weeks); trial specifics NR | EO; stance NR |
| Kim 2022 | Nintendo® Wii Balance Board | 100 | NR | NR | Sway length, Sway velocity, Weight‑symmetry index | Low‑pass 10 Hz | 3 × 30 s (3‑min rest) | EO; feet natural on WBB |
| Conte 2019 | AMTI force plates (with Vicon capture) | 1000 | NR | Vicon Nexus 1.7.1; Vicon Workstation (COM/COP calc) | COM–COP distance (AP); RMS during obstacle crossing | NR | Obstacle‑crossing stride (variable) | Barefoot walking; EO |
| Inoue 2019 | Kinetogravicorder G‑7100 (BEAR system) | NR | NR | NR | Max COP excursion (LR, AP) | NR | NR (robotic sessions 18 min) | EO; shoulder‑width stance |
| Julia‑Sanchez 2019 | Nintendo® Wii Balance Board on foam | 50 | NR | NR | ML COP scaling exponent (fractal); (area/length/velocity no change) | Savitzky–Golay (order 3, length 7) | 30 s | EO; foam surface |
| Bruyneel 2022 | kinetools 2015 (Kicarre); SP4C3‑MR transducers | 100 | NR (signals filtered; hardware specs reported) | NR | Path length (total/AP/ML), Ellipse area (95%), Mean/Max velocity; SEM/MDC reported | Butterworth 5th‑order, 45 Hz | 10 s (unstable sitting test) | Unstable sitting (seesaw); EO |
| Kwon 2024 | AMTI BP400600 (400×600 mm) | NR | NR | BioAnalysis v2.2 | Sway length, Sway velocity, Area 95% | NR | 60 s | EO/EC; stance NR (comfortable) |
| Lee 2023 | GB300 (Metitur, Finland) | 50 | NR | NR | Velocity moment; AP/ML sway speeds | NR | 30 s × 3 trials | EO/EC; stance NR |
| Ghrouz 2023 | NR (firm and foam surfaces used) | NR | NR | SPSS (stats); platform SW NR | Balance Index; BBS (no explicit COP metrics reported) | NR | 30 s (Romberg tests) | EO/EC; firm/foam |
