# Supplementary material for: Modifying center of pressure to reduce fall risk in adult stroke survivors: a scoping review
Source: Front Neurol. 2026 Apr 23;17:1773299. doi: 10.3389/fneur.2026.1773299 (PMC13149131; doi:10.3389/fneur.2026.1773299)
Supplement: Supplementary file 5 [file Table_5.docx]

**Supplementary File S4. Full Database Search Strategies**

Search date: 1 June 2025

Limits applied across databases: humans; adults (≥18 years); English language; publication date from January 1, 2015, to June 1, 2025; no geographic restrictions.

Note: Database syntax differs by platform; the strategies below use database-specific operators and field tags to implement the same core concepts.

**1) PubMed**

*((("Stroke"[Mesh] OR stroke*[tiab] OR poststroke[tiab] OR post-stroke[tiab] OR "cerebrovascular accident*"[tiab] OR CVA[tiab]))*

*AND (("center of pressure"[tiab] OR "centre of pressure"[tiab] OR COP[tiab] OR "postural sway"[tiab] OR stabilometr*[tiab] OR "force plate"[tiab] OR "force plates"[tiab]))*

*AND ((rehabilitat*[tiab] OR "balance training"[tiab] OR "postural control"[tiab] OR "gait training"[tiab] OR "weight shift*"[tiab] OR perturbation*[tiab] OR biofeedback[tiab] OR "visual feedback"[tiab]))*

*AND ((fall*[tiab] OR "fall risk"[tiab] OR "fall prevention"[tiab] OR "falls prevention"[tiab])))*

Filters: Humans; Adult: 19+ years; English; Publication dates 2015/01/01–2025/06/01.

**2) MEDLINE (Ovid syntax)**

*1. exp Stroke/ or stroke$.ti,ab. or poststroke.ti,ab. or post-stroke.ti,ab. or cerebrovascular accident$.ti,ab. or CVA.ti,ab.*

*2. (center of pressure or centre of pressure or COP or postural sway or stabilometr$ or force plate$).ti,ab.*

*3. (rehabilitat$ or balance training or postural control or gait training or weight shift$ or perturbation$ or biofeedback or visual feedback).ti,ab.*

*4. (fall$ or fall risk or fall prevention or falls prevention).ti,ab.*

*5. 1 and 2 and 3 and 4*

*6. limit 5 to (humans and english language and yr="2015–Current" and adult)*

**3) Cochrane Library**

*(stroke OR poststroke OR "post stroke" OR "cerebrovascular accident" OR CVA):ti,ab,kw*

*AND ("center of pressure" OR "centre of pressure" OR COP OR "postural sway" OR stabilometr* OR "force plate*"):ti,ab,kw*

*AND (rehabilitat* OR "balance training" OR "postural control" OR "gait training" OR "weight shift*" OR perturbation* OR biofeedback OR "visual feedback"):ti,ab,kw*

*AND (fall* OR "fall risk" OR "fall prevention"):ti,ab,kw*

Limits: Publication year 2015–2025; Trials/Reviews searched as applicable.
